# Supplementary material for: Understanding the genetics of viral drug resistance by integrating clinical data and mining of the scientific literature
Source: Sci Rep. 2022 Aug 25;12:14476. doi: 10.1038/s41598-022-17746-3 (PMC9403226; doi:10.1038/s41598-022-17746-3)
Supplement: Supplementary file 1 — Supplementary Information. [file 41598_2022_17746_MOESM1_ESM.pdf]

Supplementary Information

Understanding the Genetics of Viral Drug Resistance by Integrating Clinical Data and Mining of the Scientific Literature

An Goto[1], Raul Rodriguez-Esteban[2], Sebastian H. Scharf [2] & Garrett M. Morris[1*]

[1]Oxford Protein Informatics Group, Department of Statistics, University of Oxford, 24-29 St Giles', Oxford, OX1 3LB, U.K. [2]Roche Innovation Center Basel, Basel, Switzerland.

*Correspondence: garrett.morris@stats.ox.ac.uk.

Supporting Information

Appendix A: Prevalence of Resistance Mutations in the Clinical Study

Supplementary Figure S1: The distribution of the number of amino acid mutations mentioned in both clinical study and the literature for a specific patient……………………………………4

Supplementary Figure S2: The distribution of the number of nucleotide mutations mentioned in both clinical study and the literature for a specific patient………………………………………4

Appendix B: Counts of Mutations in Patients in the Clinical Study

Supplementary Figure S3-1: Distribution of the counts of mutations common between the clinical study and the scientific literature……………………………………………………..5

Supplementary Figure S3-2: Top 10 patients in the clinical study who had the most mutations appearing in the literature…………………………………………..……………………..5

Appendix C: Mutation Hotspots for HBV Genotypes in the Clinical Study

Supplementary Figure S4(a): Hotspot map for HBV genotype A polymerase for amino acid variants (reference sequence: AF090842) ……………………………………………………..6

Supplementary Figure S4(b): Hotspot map for HBV genotype A reverse transcriptase (RT) for amino acid variants (reference sequence: AF090842)……………………………………………7

Supplementary Figure S4(c): Hotspot map for HBV genotype A gene X for amino acid variants (reference sequence: AF090842)…………………………………………………………...8

Supplementary Figure S4(d): Hotspot map for HBV genotype A precore (gene C) for amino acid variants (reference sequence: AF090842)………………………………………………..8

Supplementary Figure S4(e): Hotspot map for HBV genotype A core (gene C) for amino acid variants (reference sequence: AF090842) ……………………………………………………9

Supplementary Figure S4(f): Hotspot map for HBV genotype A PreS1 (gene S) for amino acid variants (reference sequence: AF090842)..…………………………………………………9

Supplementary Figure S4(g): Hotspot map for HBV genotype A PreS2 (gene S) for amino acid variants (reference sequence: AF090842)……………………………………………………10

Supplementary Figure S4(h): Hotspot map for HBV genotype A HBsAg (gene S) for amino acid variants (reference sequence: AF090842)…………………………………………………11

Supplementary Figure S5(a): Hotspot map for HBV genotype B polymerase for amino acid variants (reference sequence: AB033554)……………………………………………………12

Supplementary Figure S5(b): Hotspot map for HBV genotype B reverse transcriptase (RT) for amino acid variants (reference sequence: AB033554). ………………………………………12

Supplementary Figure S5(c): Hotspot map for HBV genotype B PreS1 (gene S) for amino acid variants (reference sequence: AB033554)..…………………………………………………...13

Supplementary Figure S5(d): Hotspot map for HBV genotype B PreS2 (gene S) for amino acid variants (reference sequence: AB033554)..………………………………………………..13

Supplementary Figure S5(e): Hotspot map for HBV genotype B HBsAg (gene S) for amino acid variants (reference sequence: AB033554)...…………………………………………………14

Supplementary Figure S5(f): Hotspot map for HBV genotype B gene X for amino acid variants (reference sequence: AB033554)….……………………………………………………….14

Supplementary Figure S5(g): Hotspot map for HBV genotype B precore (gene C) for amino acid variants (reference sequence: AB033554)...15

Supplementary Figure S5(h): Hotspot map for HBV genotype B core (gene C) for amino acid variants (reference sequence: AB033554)...15

Supplementary Figure S6(a): Hotspot map for HBV genotype C polymerase for amino acid variants (reference sequence: AB033556)...16

Supplementary Figure S6(b): Hotspot map for HBV genotype C reverse transcriptase (RT) for amino acid variants (reference sequence: AB033556)...16

Supplementary Figure S6(c): Hotspot map for HBV genotype C gene PreS1 (gene S) for amino acid variants (reference sequence: AB033556)...17

Supplementary Figure S6(d): Hotspot map for HBV genotype C PreS2 (gene S) for amino acid variants (reference sequence: AB033556)...17

Supplementary Figure S6(e): Hotspot map for HBV genotype C HBsAg (gene S) for amino acid variants (reference sequence: AB033556)...18

Supplementary Figure S6(f): Hotspot map for HBV genotype C gene X for amino acid variants (reference sequence: AB033556)...19

Supplementary Figure S6(g): Hotspot map for HBV genotype C precore (gene C) for amino acid variants (reference sequence: AB033556)...19

Supplementary Figure S6(h): Hotspot map for HBV genotype C core (gene C) for amino acid variants (reference sequence: AB033556)...20

Supplementary Figure S7(a): Hotspot map for HBV genotype D polymerase for amino acid variants (reference sequence: AF121240)...20

Supplementary Figure S7(b): Hotspot map for HBV genotype D reverse transcriptase (RT) for amino acid variants (reference sequence: AF121240)...21

Supplementary Figure S7(c): Hotspot map for HBV genotype D PreS1 (gene S) for amino acid variants (reference sequence: AF121240)...22

Supplementary Figure S7(d): Hotspot map for HBV genotype D PreS2 (gene S) for amino acid variants (reference sequence: AF121240)...22

Supplementary Figure S7(e): Hotspot map for HBV genotype D HBsAg (gene S) for amino acid variants (reference sequence: AF121240)...23

Supplementary Figure S7(f): Hotspot map for HBV genotype D gene X for amino acid variants (reference sequence: AF121240)...24

Supplementary Figure S7(g): Hotspot map for HBV genotype D precore (gene C) for amino acid variants (reference sequence: AF121240)...24

Supplementary Figure S7(h): Hotspot map for HBV genotype D core (gene C) for amino acid variants (reference sequence: AF121240)...25

Supplementary Figure S8(a): Hotspot map for HBV genotype E polymerase for amino acid variants (reference sequence: AB032431) 25

Supplementary Figure S8(b): Hotspot map for HBV genotype D reverse transcriptase (RT) for amino acid variants (reference sequence: AB032431)...26

Supplementary Figure S8(c): Hotspot map for HBV genotype E gene PreS1 (gene S) for amino acid variants (reference sequence: AB032431)....……………………………………………26

Supplementary Figure S8(d): Hotspot map for HBV genotype E PreS2 (gene S) for amino acid variants (reference sequence: AB032431)………..……………………………………..27

Supplementary Figure S8(e): Hotspot map for HBV genotype E HBsAg (gene S) for amino acid variants (reference sequence: AB032431)..…………………………………………...27

Supplementary Figure S8(f): Hotspot map for HBV genotype E gene X for amino acid variants (reference sequence: AB032431)..………..…………………………………………..28

Supplementary Figure S8(g): Hotspot map for HBV genotype E precore (gene C) for amino acid variants (reference sequence: AB032431)..…………………………………………...28

Supplementary Figure S8(h): Hotspot map for HBV genotype E core (gene C) for amino acid variants (reference sequence: AB032431). ………..……………………………………29

Supplementary Figure S9: Hotspot map for HBV genotype A for nucleotide variants..………29

Supplementary Figure S10: Hotspot map for HBV genotype B for nucleotide variants..……...30

Supplementary Figure S11: Hotspot map for HBV genotype C for nucleotide variants..……...30

Supplementary Figure S12: Hotspot map for HBV genotype D for nucleotide variants..……...31

Supplementary Figure S13: Hotspot map for HBV genotype E for nucleotide variants.……....32

Supplementary Table S1. Summary of hotspots for HBV genotype A for amino acid and nucleotide variants……………………………………………………………………………32

Supplementary Table S2. Summary of hotspots for HBV genotype B for amino acid and nucleotide variants……………………………………………………………………………33

Supplementary Table S3. Summary of hotspots for HBV genotype C for amino acid and nucleotide variants……………………………………………………………………………33

Supplementary Table S4. Summary of hotspots for HBV genotype D for amino acid and nucleotide variants……………………………………………………………………………34

Supplementary Table S5. Summary of hotspots for HBV genotype E for amino acid and nucleotide variants……………………………………………………………………………34

Supplementary Figure S14. Scatter plot to represent the count of a particular mutation found in the clinical study and whether any papers referred to that mutation (Yes: 1; No: 0) for amino acids…………………………………………………………………………………..35

Supplementary Figure S15. Scatter plot to represent the count of a particular mutation found in the clinical study and whether any papers referred to that mutation (Yes: 1; No: 0) for nucleotides………..……………………………………………………………………..35

Appendix D: Probability of Drug-Resistance Mutations in the Literature and Clinical Study Counts

Supplementary Note S1: Linear model between the probability of appearing in the literature as drug-resistance-related and clinical study count…………………………………….…..36

Appendix A: Prevalence of Resistance Mutations in the Clinical Study

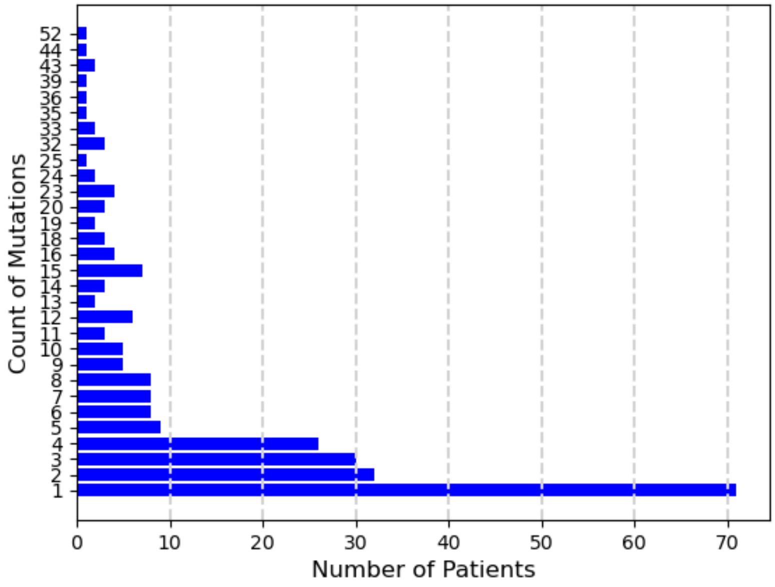

Supplementary Figure S1: The distribution of the number of amino acid mutations mentioned in both clinical study and the literature for a specific patient. The counts are taken from the number of times unique mutations common to literature and the clinical study appeared in the clinical data.

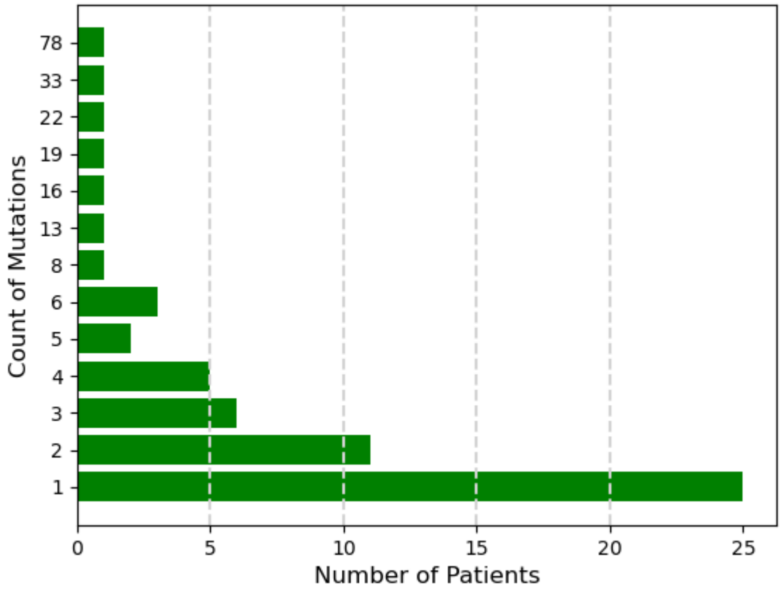

Supplementary Figure S2: The distribution of the number of nucleotide mutations mentioned in both clinical study and the literature for a specific patient. The counts are taken from the number of times unique mutations common to literature and the clinical study appeared in the clinical data.

Appendix B: Counts of Mutations in Patients in the Clinical Study

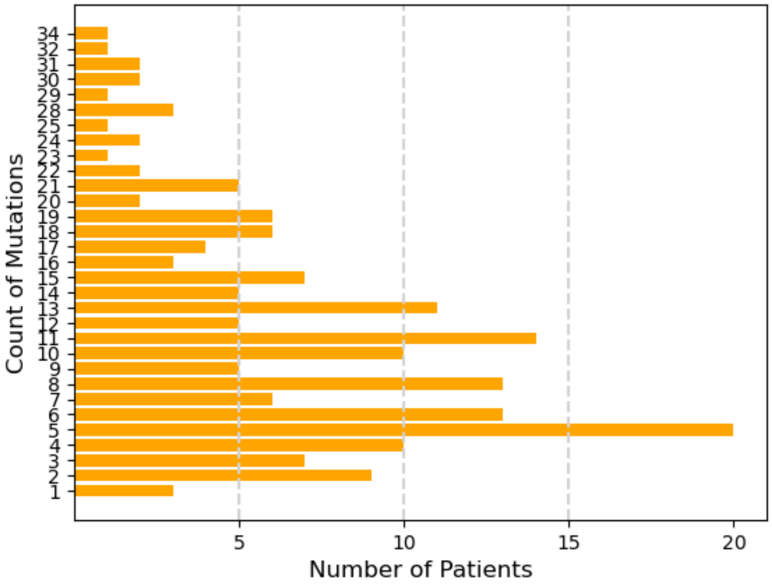

Supplementary Figure S3-1: Distribution of the counts of mutations common between the clinical study and the scientific literature. The counts for each of the patients are taken from the number of times the common mutations between the clinical data and the literature appeared in the clinical data.

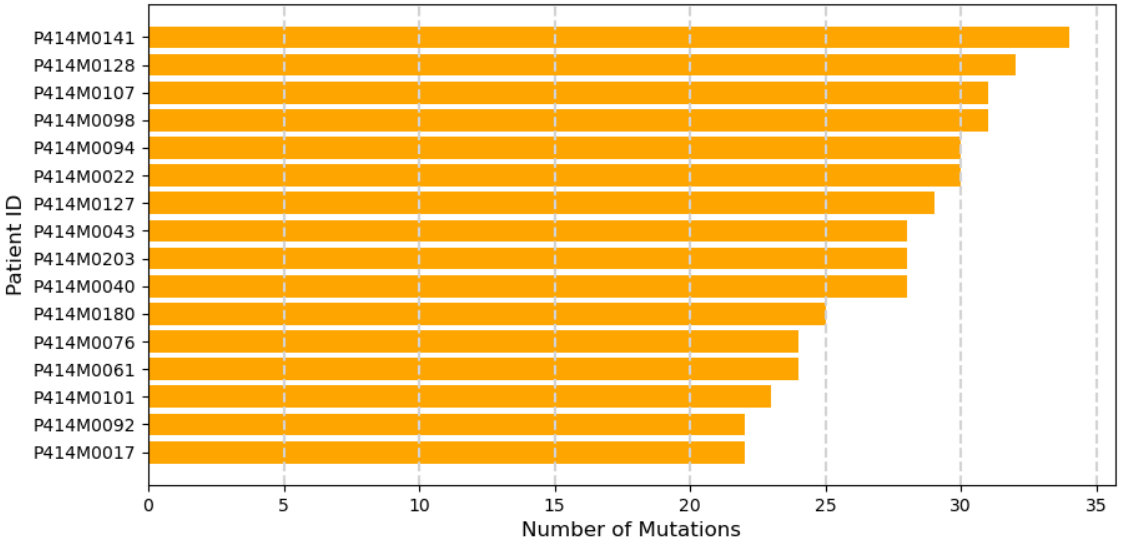

Supplementary Figure S3-2: Top 10 patients in the clinical study who had the most mutations appearing in the literature.

Appendix C: Mutation Hotspots for HBV Genotypes in the Clinical Study

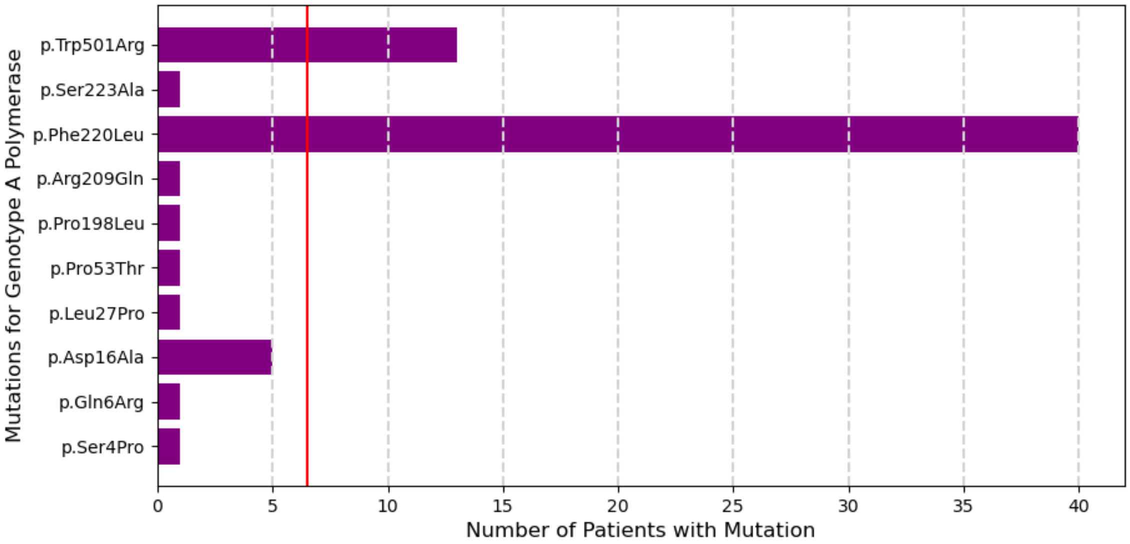

Supplementary Figure S4(a): Mutation hotspot map for HBV genotype A polymerase for amino acid variants. The counts for each mutation are based on the number of times a particular mutation common between the scientific literature and the clinical study appeared in the clinical data. The red line represents the average count of patients with a mutation for genotype A polymerase, which is 6.5 patients. The order of the mutations in the bar chart is based on the position of the nucleotides in the genes for genotype A according to the GenBank accession code AF090842.

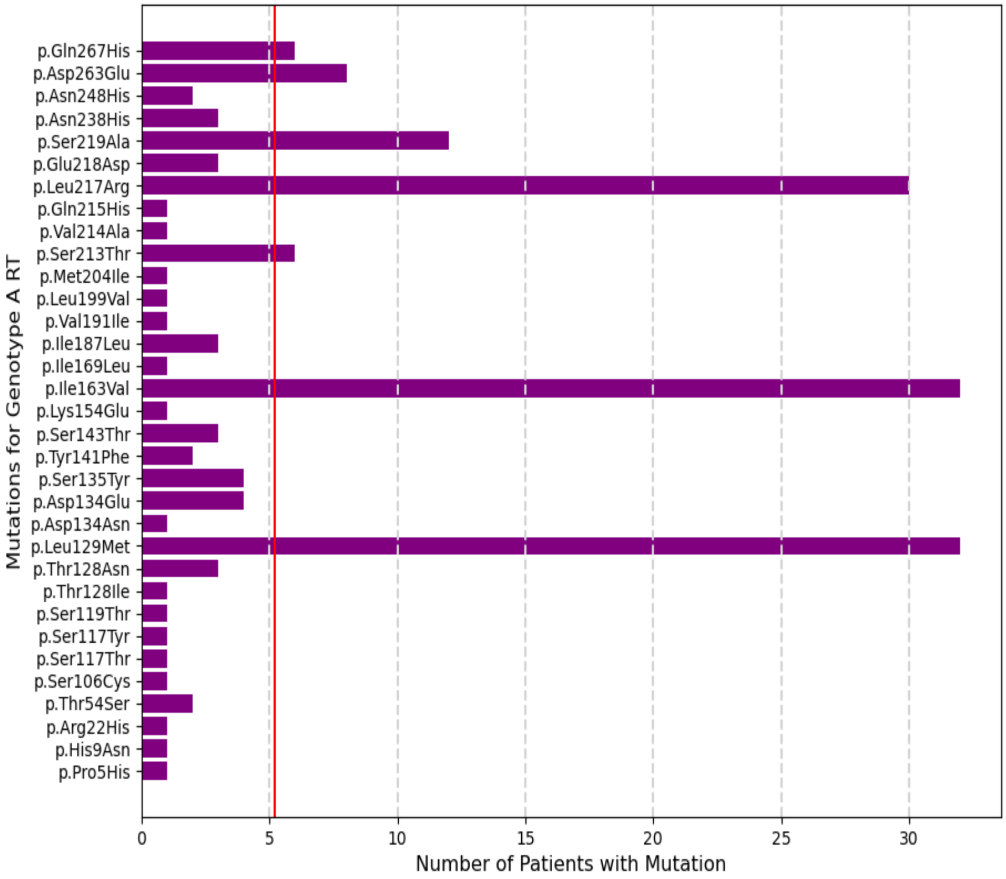

Supplementary Figure S4(b): Mutation hotspot map for HBV genotype A reverse transcriptase (RT) for amino acid variants. The counts for each mutation are based on the number of times a particular mutation common between the scientific literature and the clinical study appeared in the clinical data. The red line represents the average count of patients with a mutation for genotype A reverse transcriptase, which is 5.18 patients. The order of the mutations in the bar chart is based on the position of the nucleotides in the genes for genotype A according to the GenBank accession code AF090842.

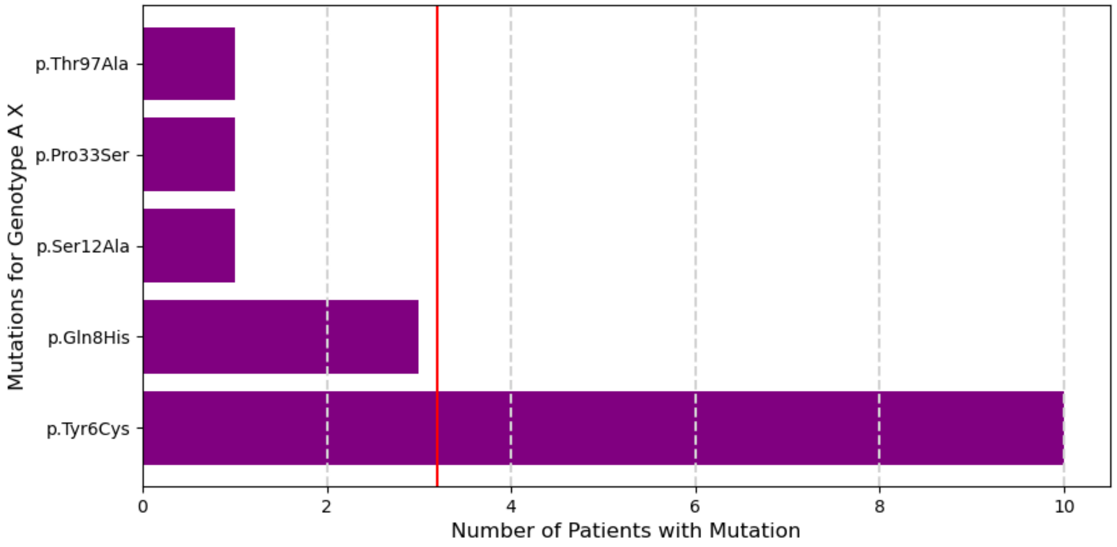

Supplementary Figure S4(c): Mutation hotspot map for HBV genotype A gene X for amino acid variants. The counts for each mutation are based on the number of times a particular mutation common between the scientific literature and the clinical study appeared in the clinical data. The red line represents the average count of patients with a mutation for genotype A gene X, which is 3.2 patients. The order of the mutations in the bar chart is based on the position of the nucleotides in the genes for genotype A according to the GenBank accession code AF090842.

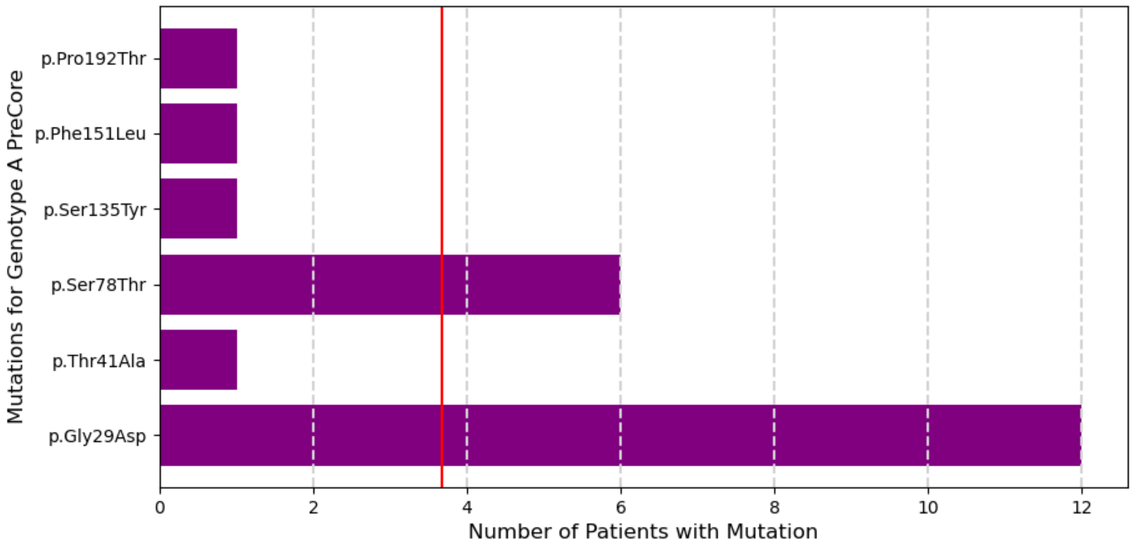

Supplementary Figure S4(d): Mutation hotspot map for HBV genotype A precore (gene C) for amino acid variants. The counts for each mutation are based on the number of times a particular mutation common between the scientific literature and the clinical study appeared in the clinical data. The red line represents the average count of patients with a mutation for genotype A precore, which is 3.67 patients. The order of the mutations in the bar chart is based on the position of the nucleotides in the genes for genotype A according to the GenBank accession code AF090842.

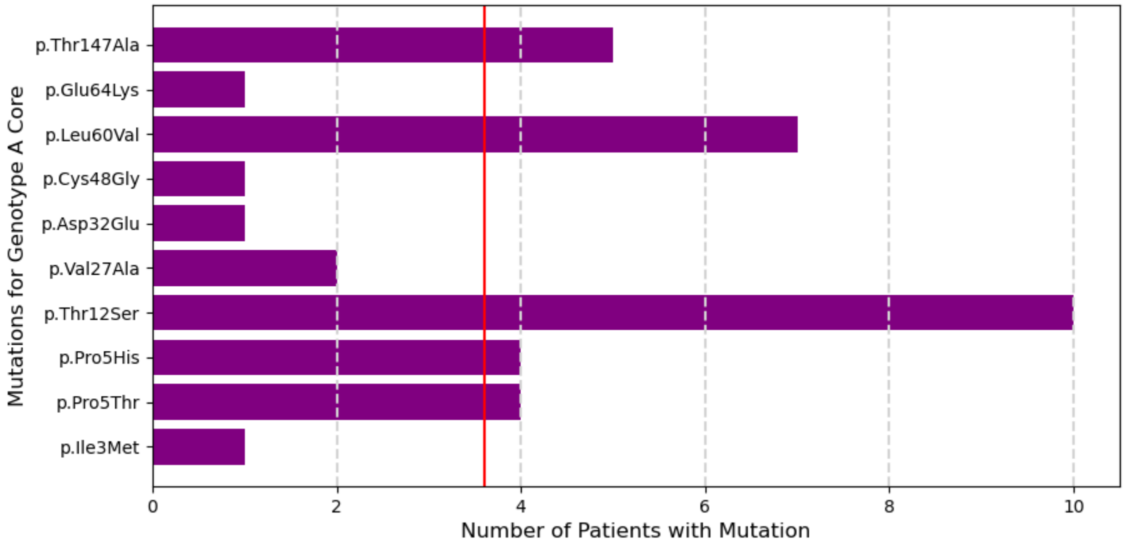

Supplementary Figure S4(e): Mutation hotspot map for HBV genotype A core (gene C) for amino acid variants. The counts for each mutation are based on the number of times a particular mutation common between the scientific literature and the clinical study appeared in the clinical data. The red line represents the average count of patients with a mutation for genotype A core, which is 3.6 patients. The order of the mutations in the bar chart is based on the position of the nucleotides in the genes for genotype A according to the GenBank accession code AF090842.

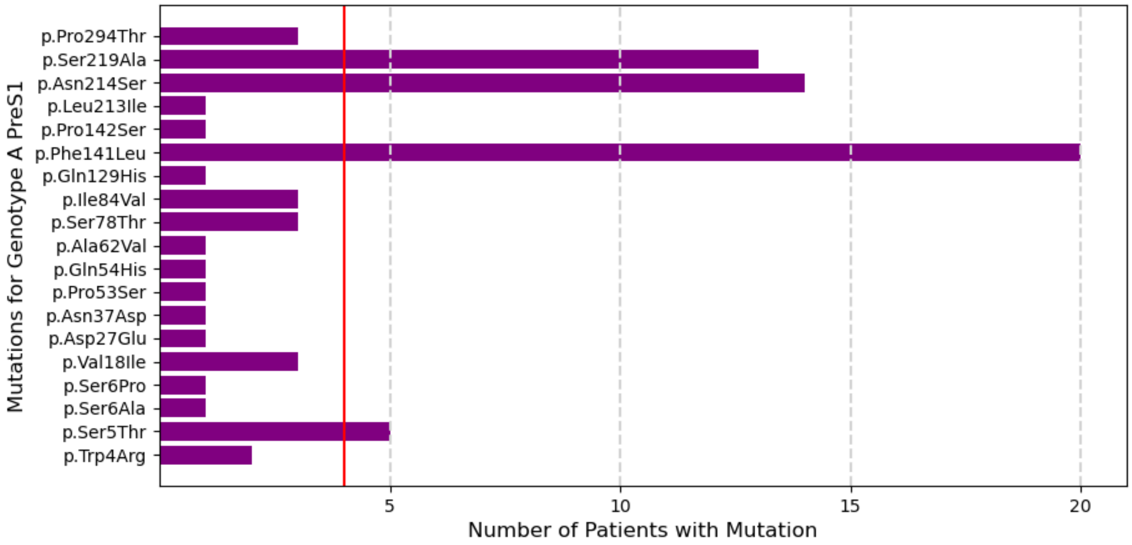

Supplementary Figure S4(f): Mutation hotspot map for HBV genotype A PreS1 (gene S) for amino acid variants. The counts for each mutation are based on the number of times a particular mutation common between the scientific literature and the clinical study appeared in the clinical data. The red line represents the average count of patients with a mutation for genotype A PreS1, which is 4 patients. The order of the mutations in the bar chart is based on the position of the nucleotides in the genes for genotype A according to the GenBank accession code AF090842.

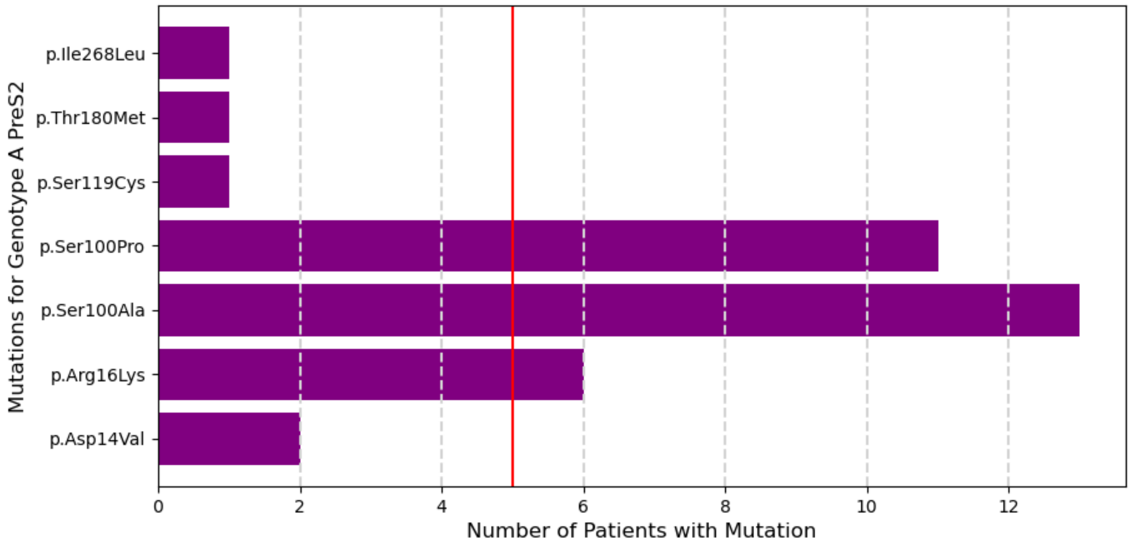

Supplementary Figure S4(g): Mutation hotspot map for HBV genotype A PreS2 (gene S) for amino acid variants. The counts for each mutation are based on the number of times a particular mutation common between the scientific literature and the clinical study appeared in the clinical data. The red line represents the average count of patients with a mutation for genotype A PreS2, which is 5 patients. The order of the mutations in the bar chart is based on the position of the nucleotides in the genes for genotype A according to the GenBank accession code AF090842.

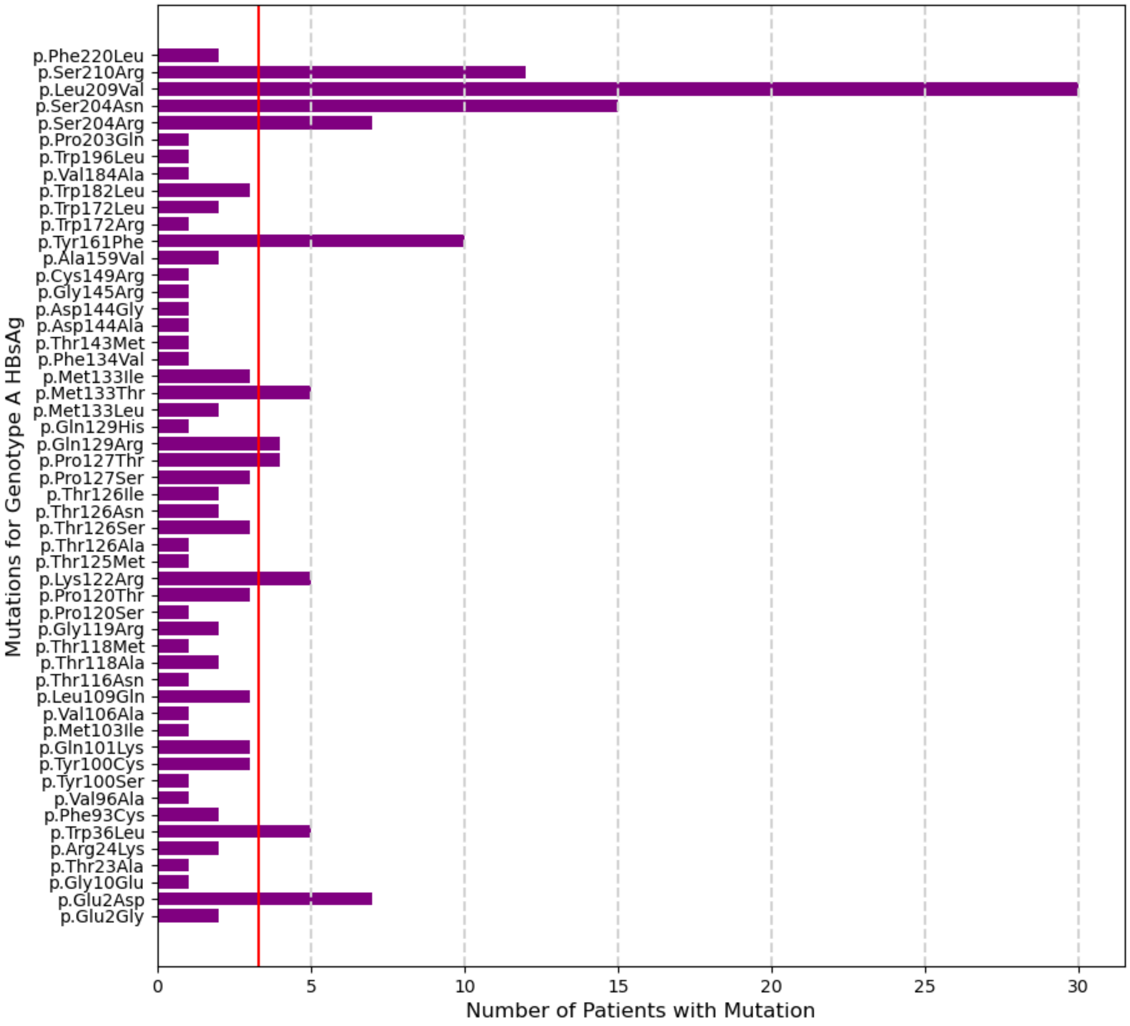

Supplementary Figure S4(h): Mutation hotspot map for HBV genotype A HBsAg (gene S) for amino acid variants. The counts for each mutation are based on the number of times a particular mutation common between the scientific literature and the clinical study appeared in the clinical data. The red line represents the average count of patients with a mutation for genotype A HBsAg, which is 3.31 patients. The order of the mutations in the bar chart is based on the position of the nucleotides in the genes for genotype A according to the GenBank accession code AF090842.

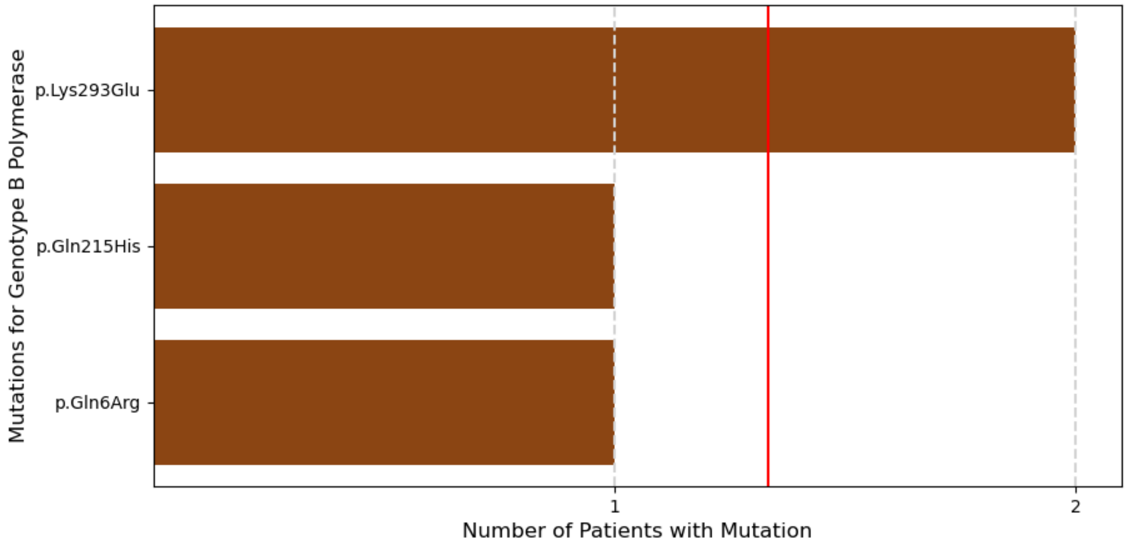

Supplementary Figure S5(a): Mutation hotspot map for HBV genotype B polymerase for amino acid variants. The counts for each mutation are based on the number of times a particular mutation common between the scientific literature and the clinical study appeared in the clinical data. The red line represents the average count of patients with a mutation for genotype B polymerase, which is 1.33 patients. The order of the mutations in the bar chart is based on the position of the nucleotides in the genes for genotype B according to the GenBank accession code AB033554.

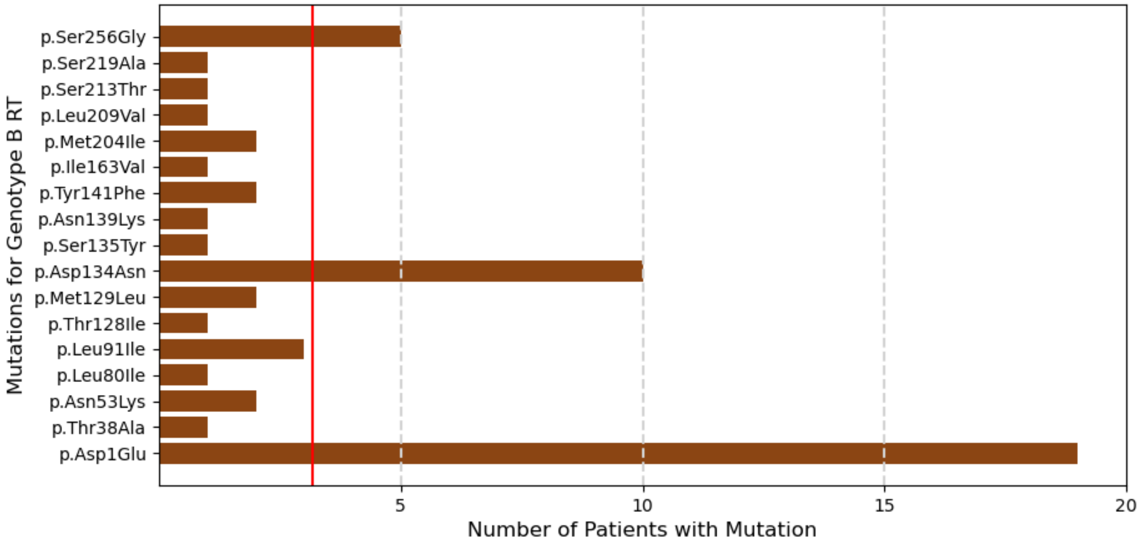

Supplementary Figure S5(b): Mutation hotspot map for HBV genotype B reverse transcriptase (RT) for amino acid variants. The counts for each mutation are based on the number of times a particular mutation common between the scientific literature and the clinical study appeared in the clinical data. The red line represents the average count of patients with a mutation for genotype B reverse transcriptase, which is 3.18 patients. The order of the mutations in the bar chart is based on the position of the nucleotides in the genes for genotype B according to the GenBank accession code AB033554.

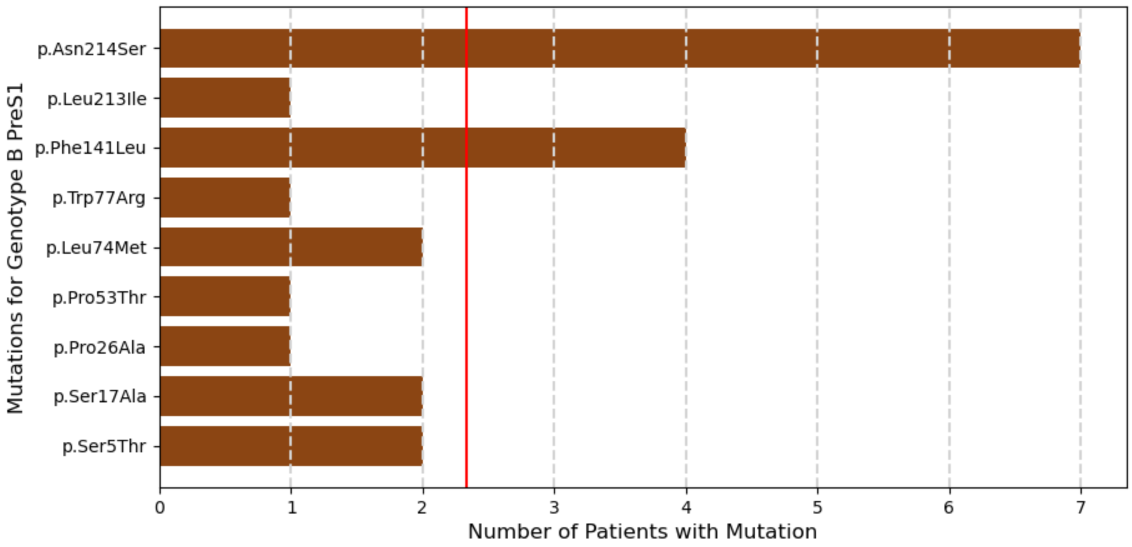

Supplementary Figure S5(c): Mutation hotspot map for HBV genotype B PreS1 (gene S) for amino acid variants. The counts for each mutation are based on the number of times a particular mutation common between the scientific literature and the clinical study appeared in the clinical data. The red line represents the average count of patients with a mutation for genotype B PreS1, which is 2.33 patients. The order of the mutations in the bar chart is based on the position of the nucleotides in the genes for genotype B according to the GenBank accession code AB033554.

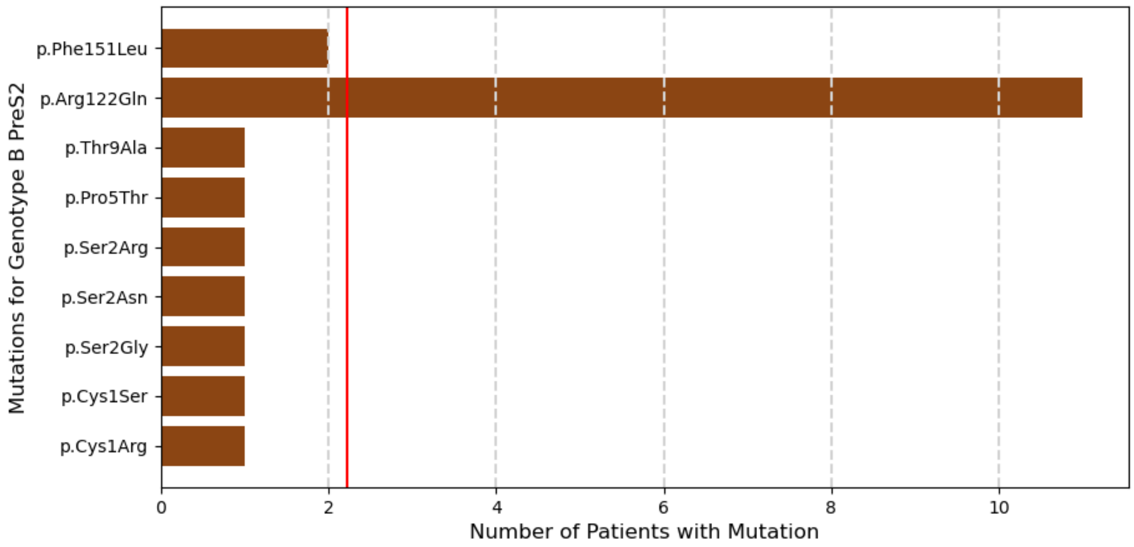

Supplementary Figure S5(d): Mutation hotspot map for HBV genotype B PreS2 (gene S) for amino acid variants. The counts for each mutation are based on the number of times a particular mutation common between the scientific literature and the clinical study appeared in the clinical data. The red line represents the average count of patients with a mutation for genotype B PreS2, which is 2.22 patients. The order of the mutations in the bar chart is based on the position of the nucleotides in the genes for genotype B according to the GenBank accession code AB033554.

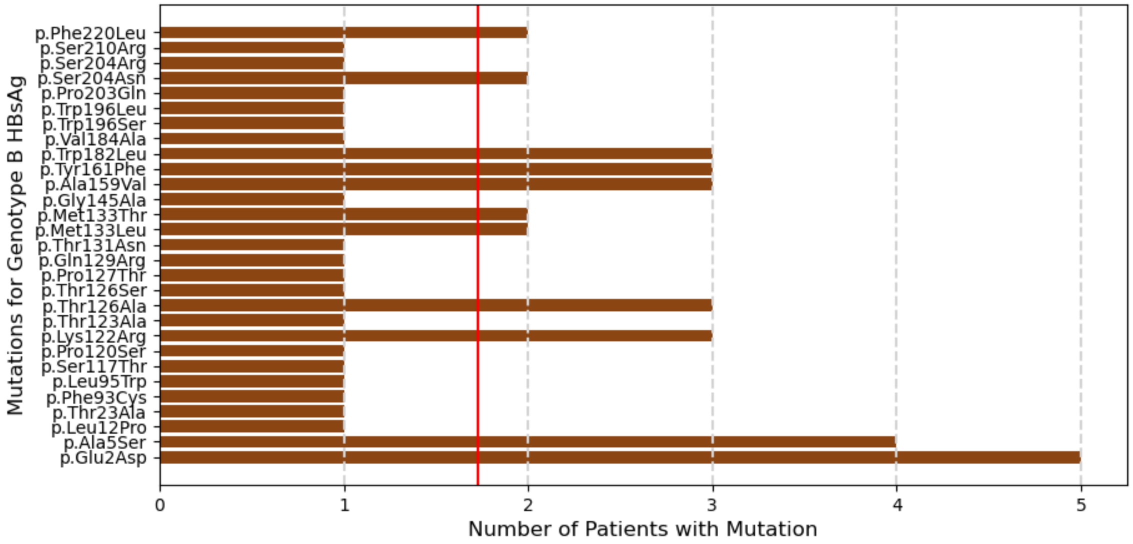

Supplementary Figure S5(e): Mutation hotspot map for HBV genotype B HBsAg (gene S) for amino acid variants. The counts for each mutation are based on the number of times a particular mutation common between the scientific literature and the clinical study appeared in the clinical data. The red line represents the average count of patients with a mutation for genotype B HBsAg, which is 1.72 patients. The order of the mutations in the bar chart is based on the position of the nucleotides in the genes for genotype B according to the GenBank accession code AB033554.

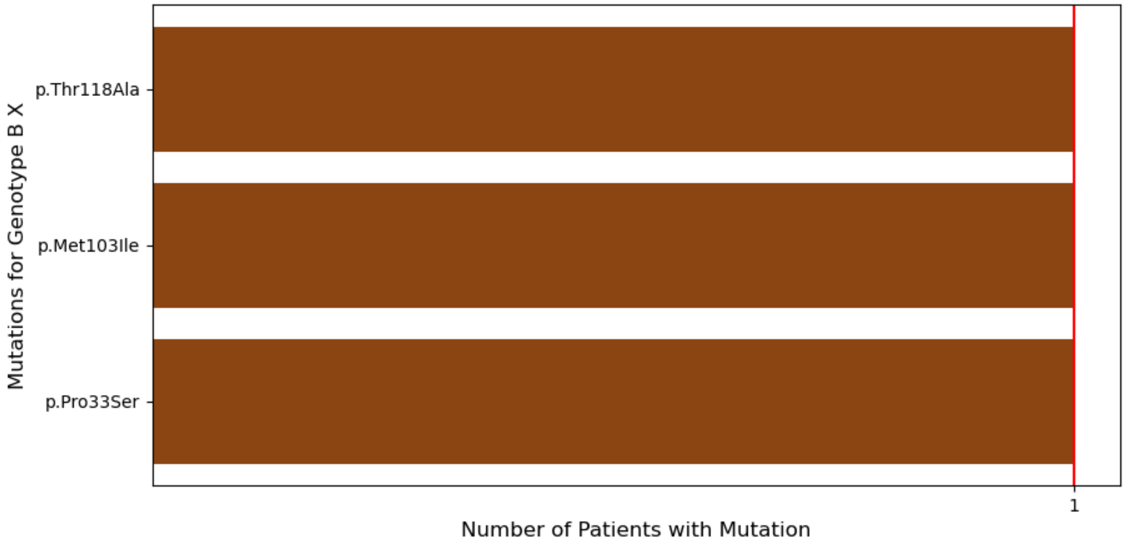

Supplementary Figure S5(f): Mutation hotspot map for HBV genotype B gene X for amino acid variants. The counts for each mutation are based on the number of times a particular mutation common between the scientific literature and the clinical study appeared in the clinical data. The red line represents the average count of patients with a mutation for genotype B gene X, which is 1 patient. The order of the mutations in the bar chart is based on the position of the nucleotides in the genes for genotype B according to the GenBank accession code AB033554.

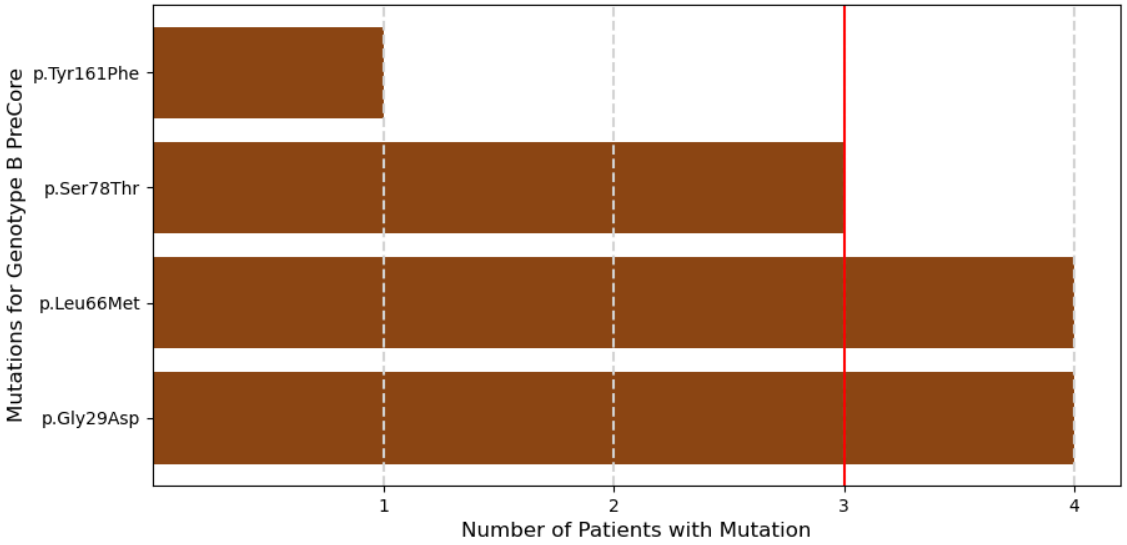

Supplementary Figure S5(g): Mutation hotspot map for HBV genotype B precore (gene C) for amino acid variants. The counts for each mutation are based on the number of times a particular mutation common between the scientific literature and the clinical study appeared in the clinical data. The red line represents the average count of patients with a mutation for genotype B precore, which is 3 patients. The order of the mutations in the bar chart is based on the position of the nucleotides in the genes for genotype B according to the GenBank accession code AB033554.

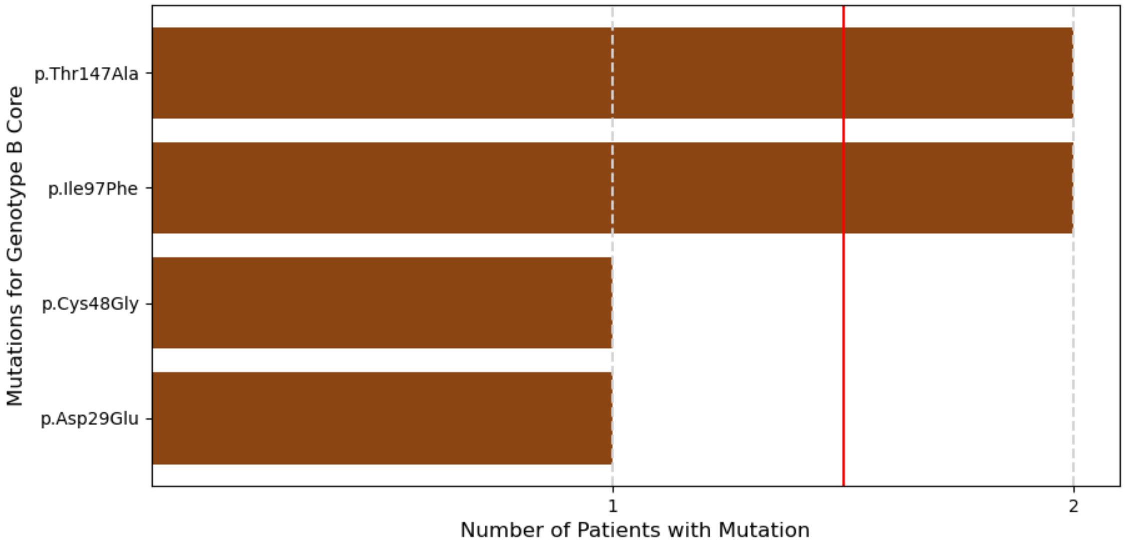

Supplementary Figure S5(h): Mutation hotspot map for HBV genotype B core (gene C) for amino acid variants. The counts for each mutation are based on the number of times a particular mutation common between the scientific literature and the clinical study appeared in the clinical data. The red line represents the average count of patients with a mutation for genotype B core, which is 1.5 patients. The order of the mutations in the bar chart is based on the position of the nucleotides in the genes for genotype B according to the GenBank accession code AB033554.

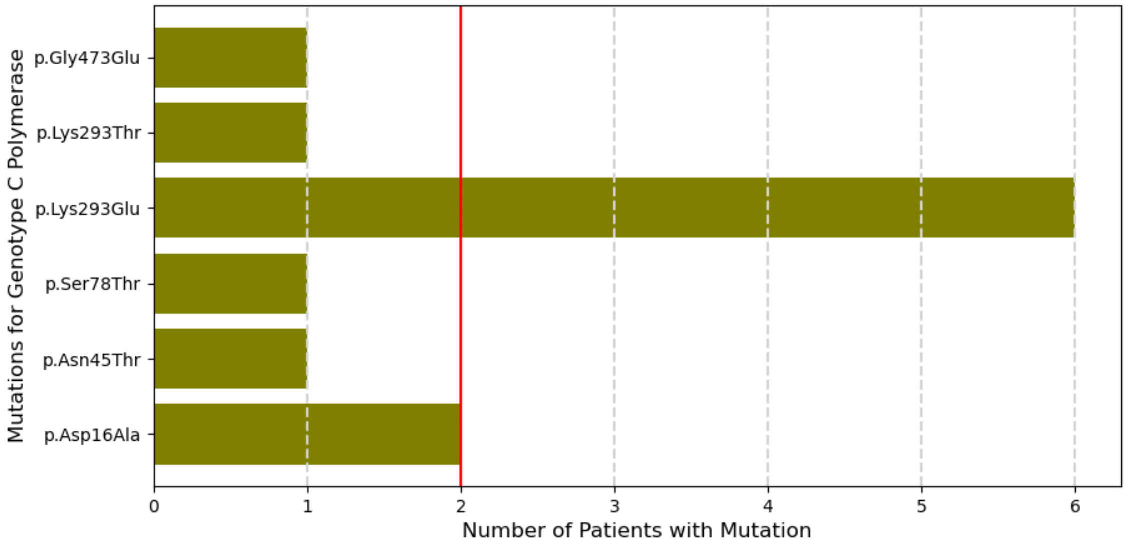

Supplementary Figure S6(a): Mutation hotspot map for HBV genotype C polymerase for amino acid variants. The counts for each mutation are based on the number of times a particular mutation common between the scientific literature and the clinical study appeared in the clinical data. The red line represents the average count of patients with a mutation for genotype C polymerase, which is 2 patients. The order of the mutations in the bar chart is based on the position of the nucleotides in the genes for genotype C according to the GenBank accession code AB033556.

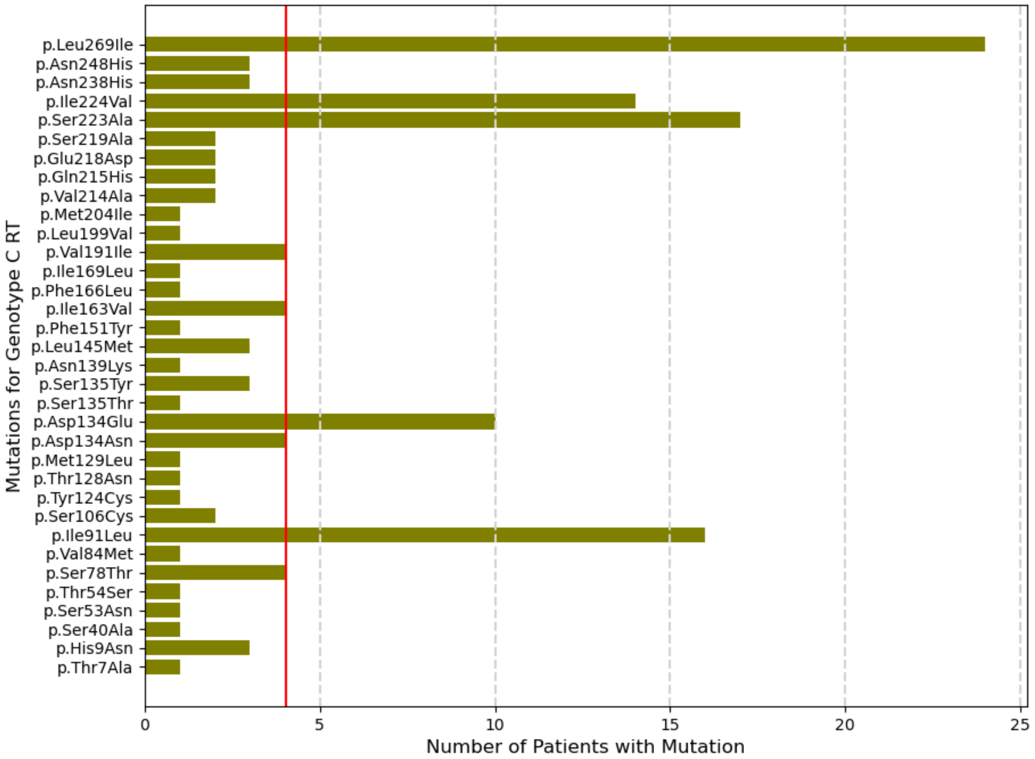

Supplementary Figure S6(b): Mutation hotspot map for HBV genotype C reverse transcriptase (RT) for amino acid variants. The counts for each mutation are based on the number of times a particular mutation common between the scientific literature and the clinical study appeared in the clinical data. The red line represents the average count of patients with a mutation for genotype C reverse transcriptase, which is 4.03

patients. The order of the mutations in the bar chart is based on the position of the nucleotides in the genes for genotype C according to the GenBank accession code AB033556.

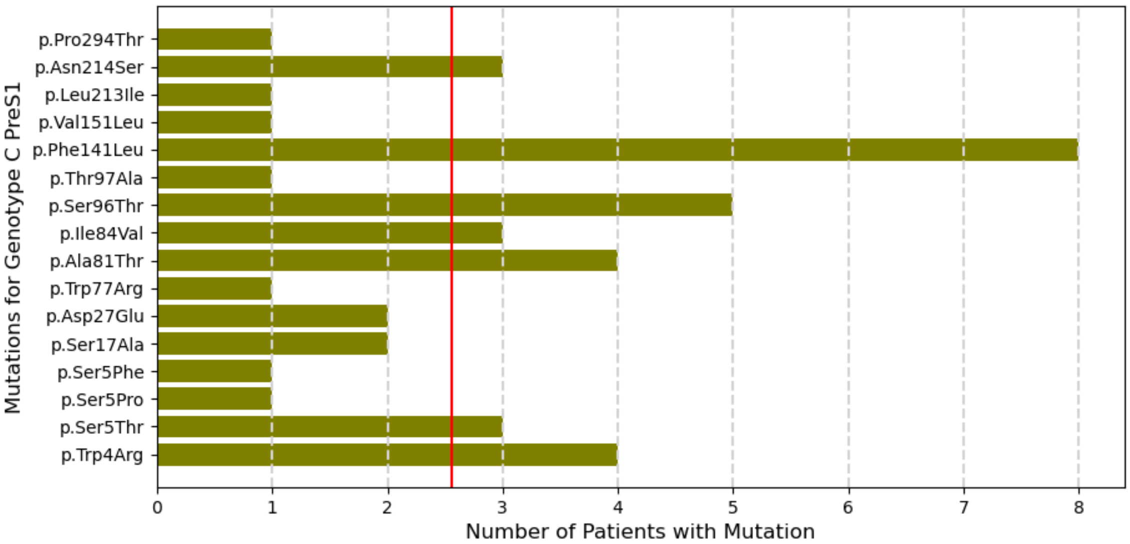

Supplementary Figure S6(c): Mutation hotspot map for HBV genotype C PreS1 (gene S) for amino acid variants. The counts for each mutation are based on the number of times a particular mutation common between the scientific literature and the clinical study appeared in the clinical data. The red line represents the average count of patients with a mutation for genotype C PreS1, which is 2.56 patients. The order of the mutations in the bar chart is based on the position of the nucleotides in the genes for genotype C according to the GenBank accession code AB033556.

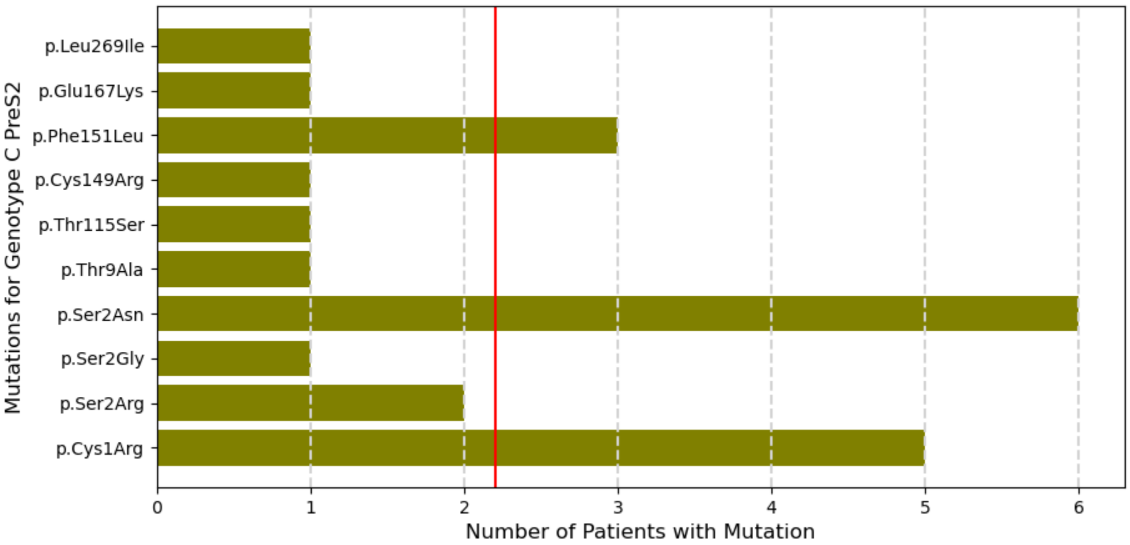

Supplementary Figure S6(d): Mutation hotspot map for HBV genotype C PreS2 (gene S) for amino acid variants. The counts for each mutation are based on the number of times a particular mutation common between the scientific literature and the clinical study appeared in the clinical data. The red line represents the average count of patients with a mutation for genotype C PreS2, which is 2.2 patients. The order of the mutations in the bar chart is based on the position of the nucleotides in the genes for genotype C according to the GenBank accession code AB033556.

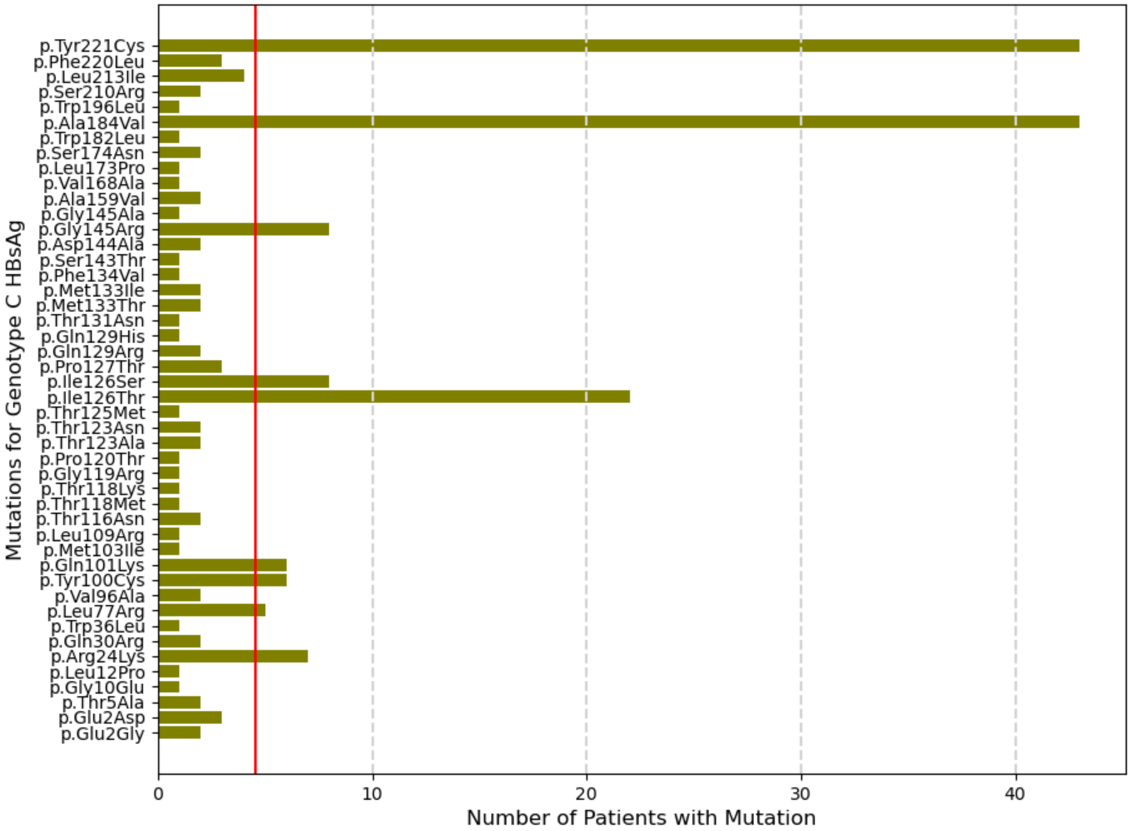

Supplementary Figure S6(e): Mutation hotspot map for HBV genotype C HBsAg (gene S) for amino acid variants. The counts for each mutation are based on the number of times a particular mutation common between the scientific literature and the clinical study appeared in the clinical data. The red line represents the average count of patients with a mutation for genotype C HBsAg, which is 4.52 patients. The order of the mutations in the bar chart is based on the position of the nucleotides in the genes for genotype C according to the GenBank accession code AB033556.

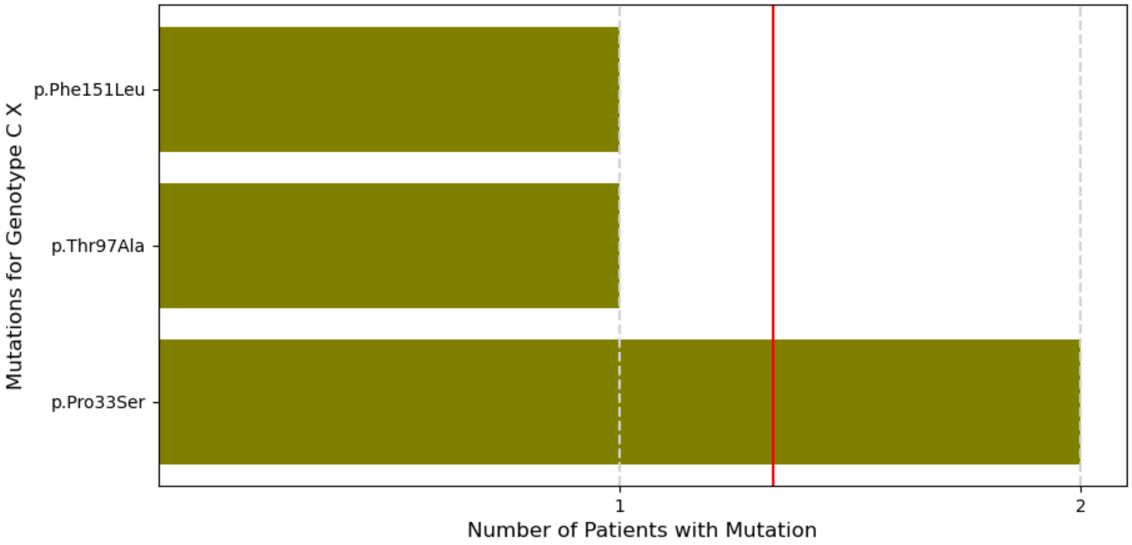

Supplementary Figure S6(f): Mutation hotspot map for HBV genotype C gene X for amino acid variants. The counts for each mutation are based on the number of times a particular mutation common between the scientific literature and the clinical study appeared in the clinical data. The red line represents the average count of patients with a mutation for genotype C gene X, which is 1.33 patients. The order of the mutations in the bar chart is based on the position of the nucleotides in the genes for genotype C according to the GenBank accession code AB033556.

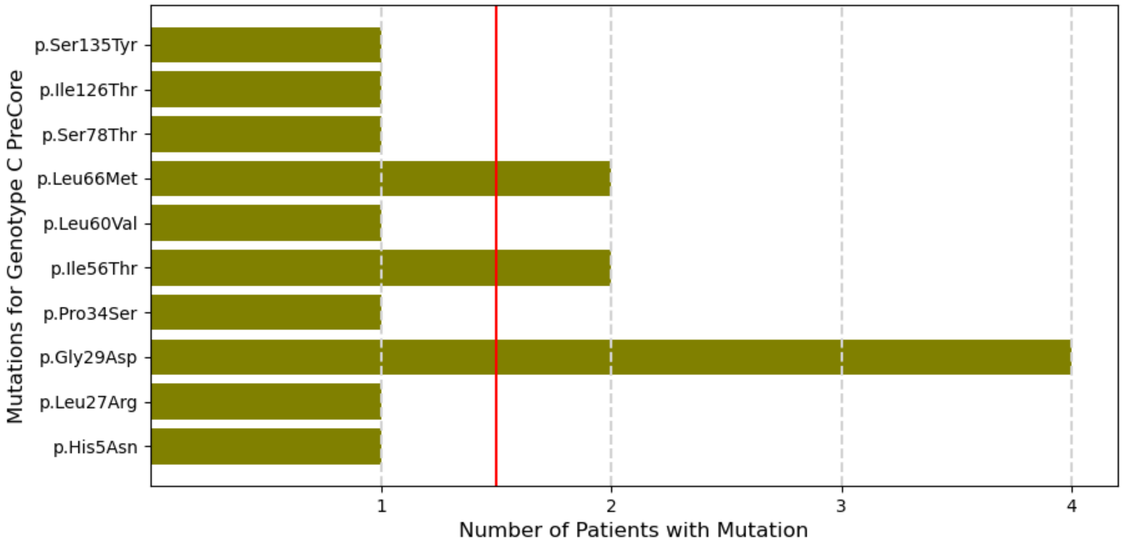

Supplementary Figure S6(g): Mutation hotspot map for HBV genotype C precore (gene C) for amino acid variants. The counts for each mutation are based on the number of times a particular mutation common between the scientific literature and the clinical study appeared in the clinical data. The red line represents the average count of patients with a mutation for genotype C precore, which is 1.5 patients. The order of the mutations in the bar chart is based on the position of the nucleotides in the genes for genotype C according to the GenBank accession code AB033556.

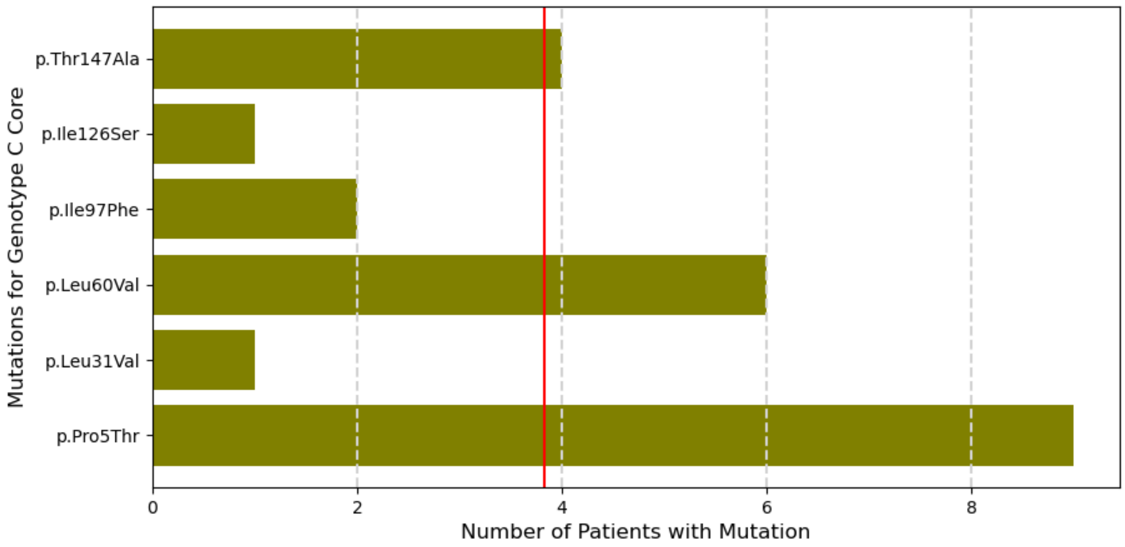

Supplementary Figure S6(h): Mutation hotspot map for HBV genotype C core (gene C) for amino acid variants. The counts for each mutation are based on the number of times a particular mutation common between the scientific literature and the clinical study appeared in the clinical data. The red line represents the average count of patients with a mutation for genotype C core, which is 3.83 patients. The order of the mutations in the bar chart is based on the position of the nucleotides in the genes for genotype C according to the GenBank accession code AB033556.

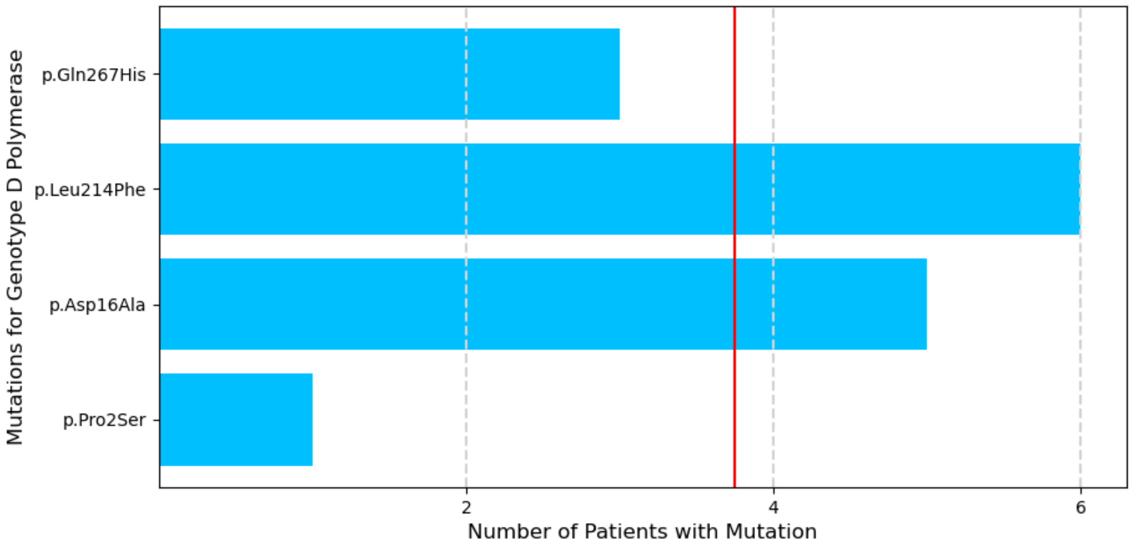

Supplementary Figure S7(a): Mutation hotspot map for HBV genotype D polymerase for amino acid variants. The counts for each mutation are based on the number of times a particular mutation common between the scientific literature and the clinical study appeared in the clinical data. The red line represents the average count of patients with a mutation for genotype D polymerase, which is 3.75 patients. The order of the mutations in the bar chart is based on the position of the nucleotides in the genes for genotype D according to the GenBank accession code AF121240.

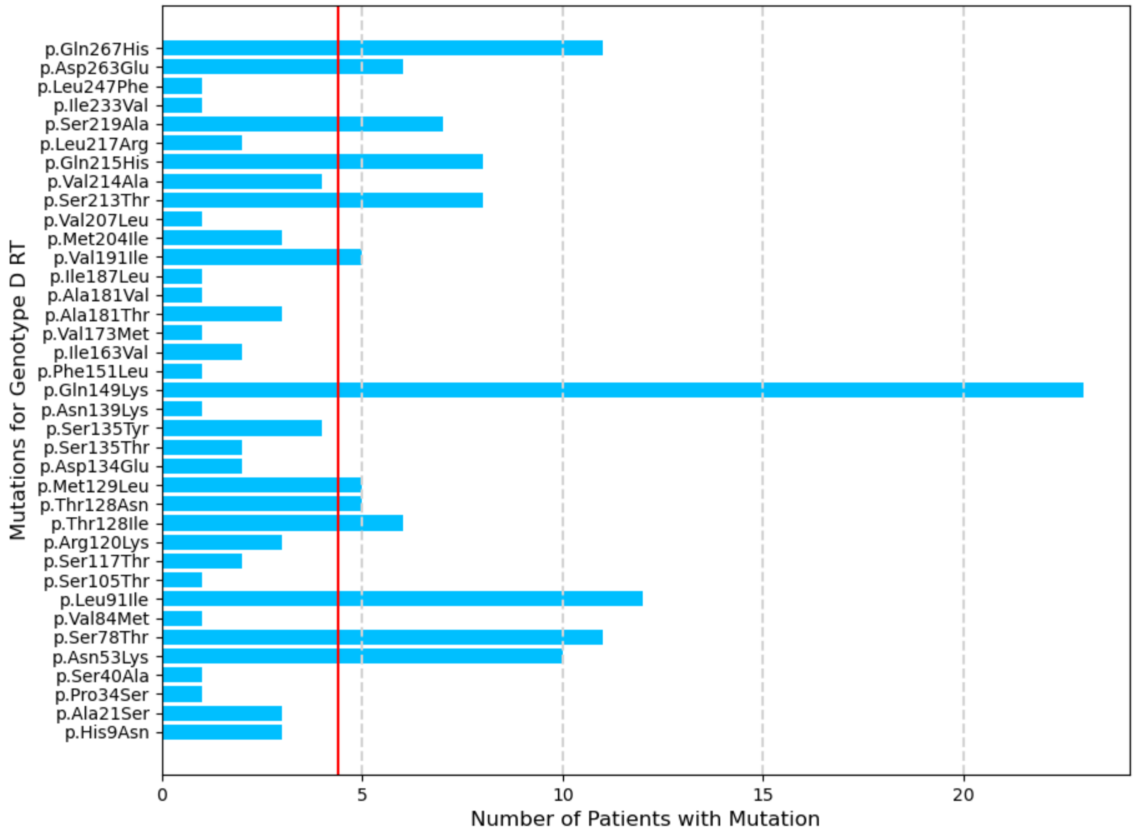

Supplementary Figure S7(b): Mutation hotspot map for HBV genotype D reverse transcriptase (RT) for amino acid variants. The counts for each mutation are based on the number of times a particular mutation common between the scientific literature and the clinical study appeared in the clinical data. The red line represents the average count of patients with a mutation for genotype D reverse transcriptase, which is 4.38 patients. The order of the mutations in the bar chart is based on the position of the nucleotides in the genes for genotype D according to the GenBank accession code AF121240.

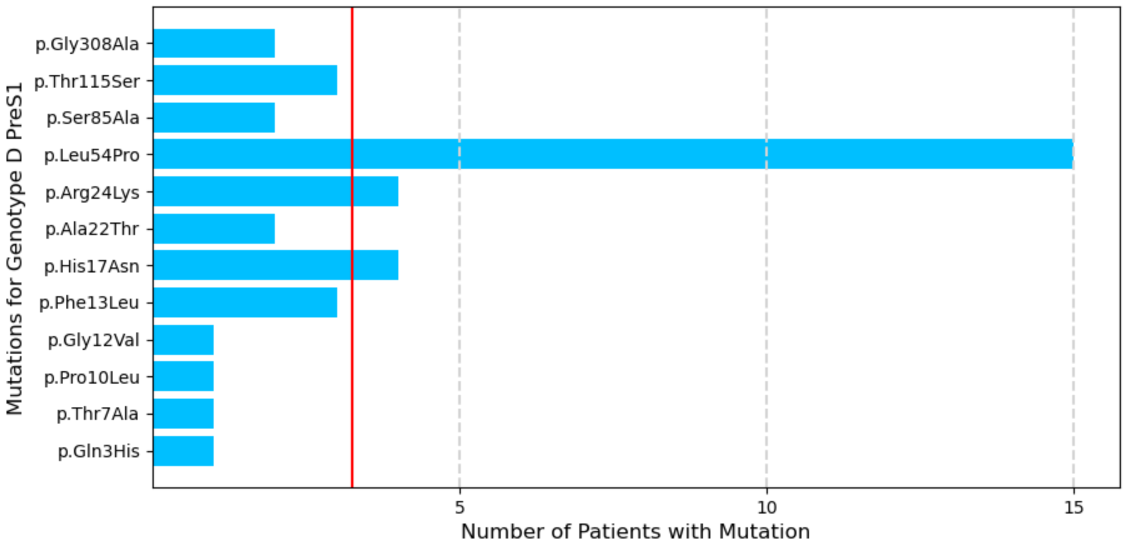

Supplementary Figure S7(c): Mutation hotspot map for HBV genotype D PreS1 (gene S) for amino acid variants. The counts for each mutation are based on the number of times a particular mutation common between the scientific literature and the clinical study appeared in the clinical data. The red line represents the average count of patients with a mutation for genotype D PreS1, which is 3.25 patients. The order of the mutations in the bar chart is based on the position of the nucleotides in the genes for genotype D according to the GenBank accession code AF121240.

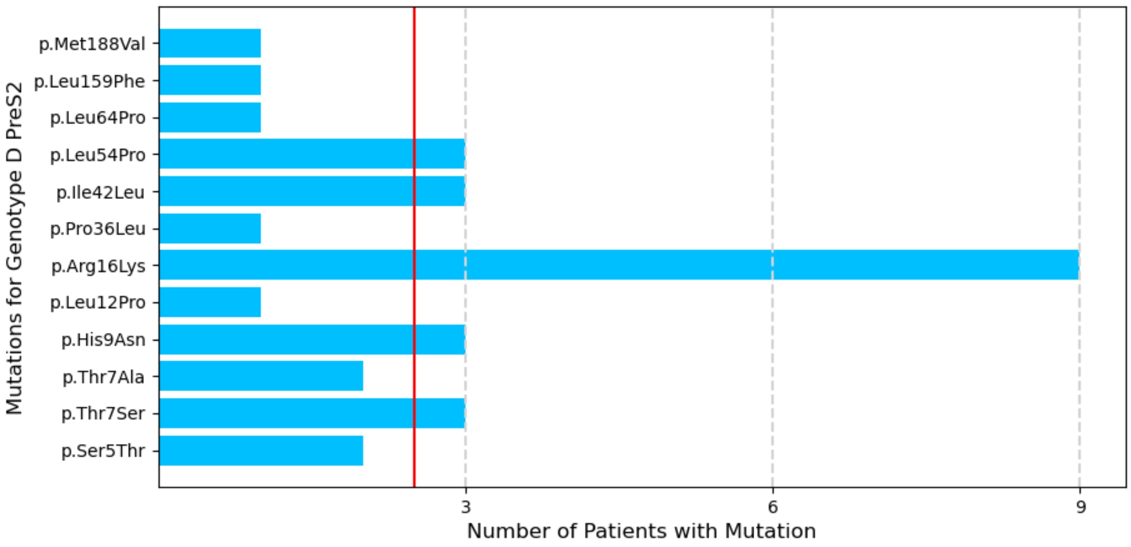

Supplementary Figure S7(d): Mutation hotspot map for HBV genotype D PreS2 (gene S) for amino acid variants. The counts for each mutation are based on the number of times a particular mutation common between the scientific literature and the clinical study appeared in the clinical data. The red line represents the average count of patients with a mutation for genotype D PreS2, which is 2.5 patients. The order of the

mutations in the bar chart is based on the position of the nucleotides in the genes for genotype D according to the GenBank accession code AF121240.

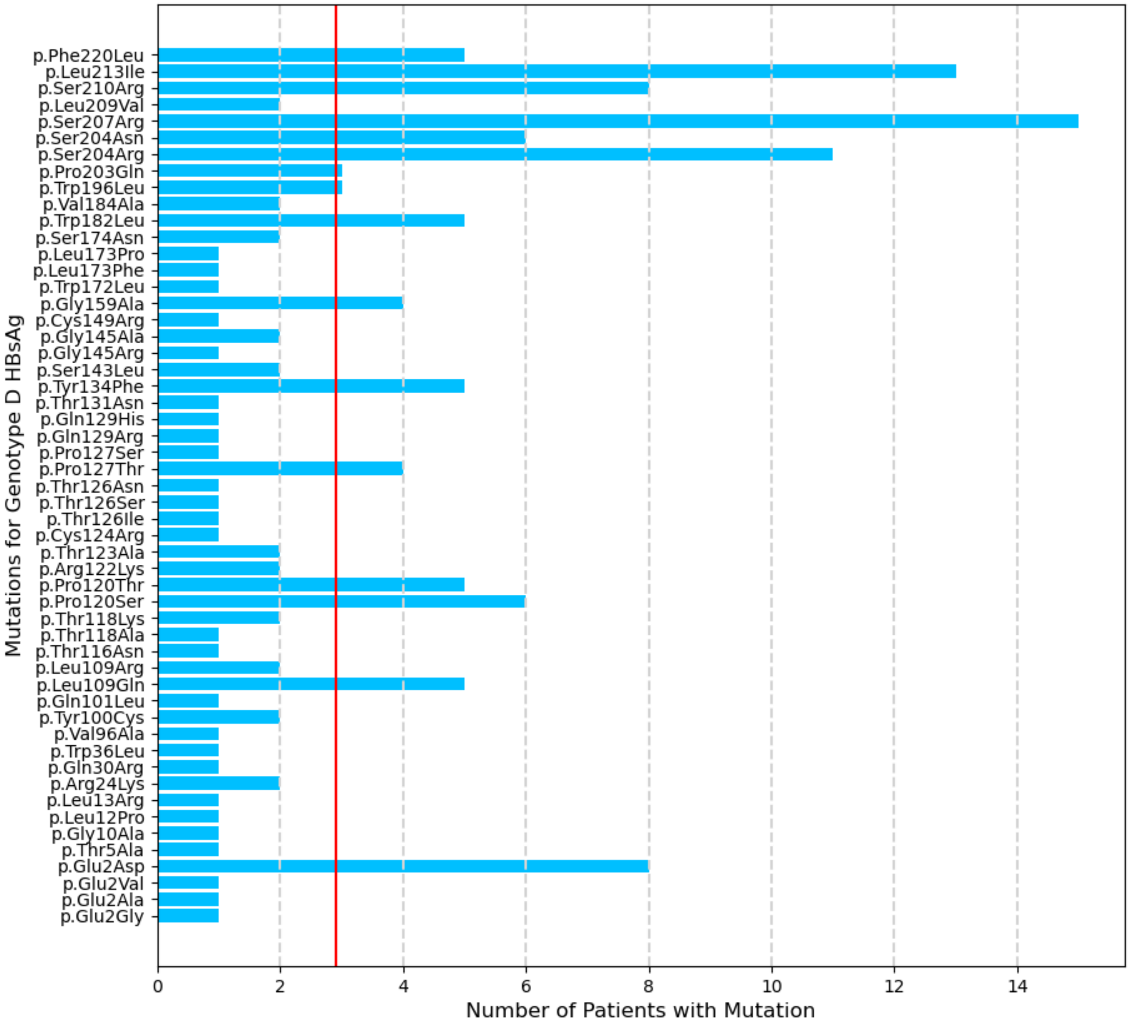

Supplementary Figure S7(e): Mutation hotspot map for HBV genotype D HBsAg (gene S) for amino acid variants. The counts for each mutation are based on the number of times a particular mutation common between the scientific literature and the clinical study appeared in the clinical data. The red line represents the average count of patients with a mutation for genotype D HBsAg, which is 2.91 patients. The order of the mutations in the bar chart is based on the position of the nucleotides in the genes for genotype D according to the GenBank accession code AF121240.

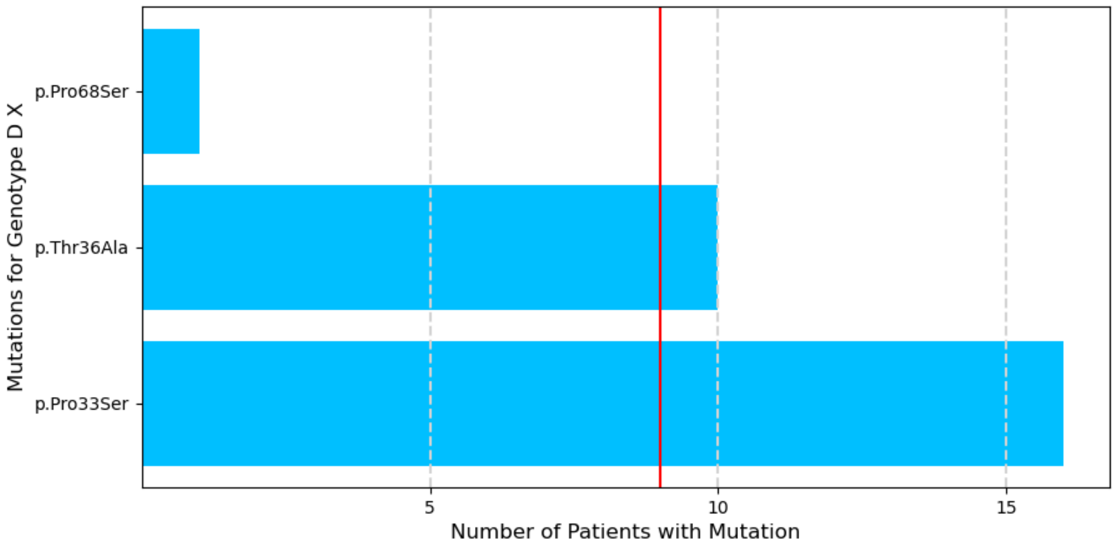

Supplementary Figure S7(f): Mutation hotspot map for HBV genotype D gene X for amino acid variants. The counts for each mutation are based on the number of times a particular mutation common between the scientific literature and the clinical study appeared in the clinical data. The red line represents the average count of patients with a mutation for genotype D gene X, which is 9 patients. The order of the mutations in the bar chart is based on the position of the nucleotides in the genes for genotype D according to the GenBank accession code AF121240.

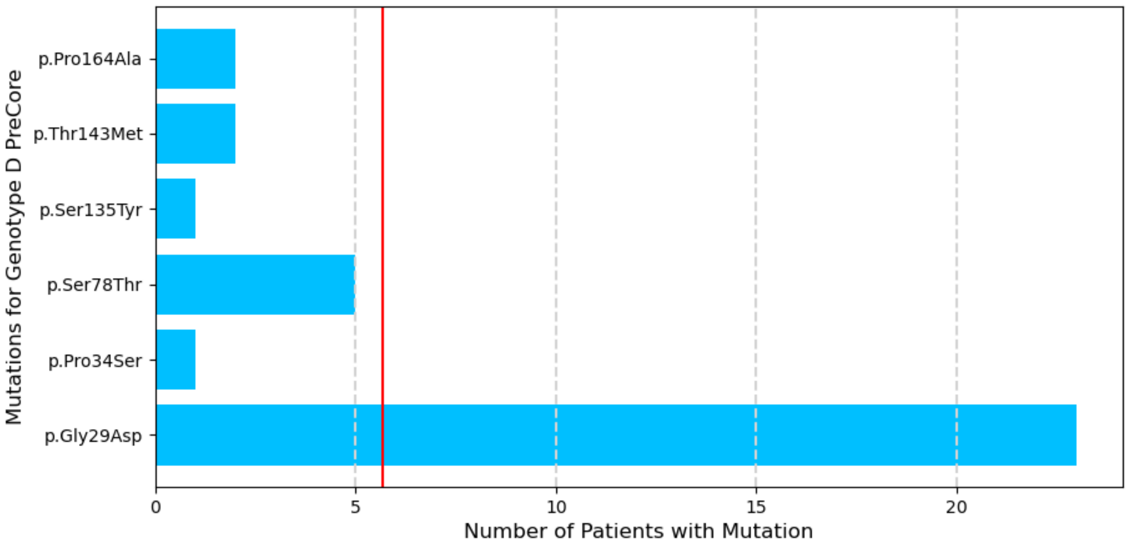

Supplementary Figure S7(g): Mutation hotspot map for HBV genotype D precore (gene C) for amino acid variants. The counts for each mutation are based on the number of times a particular mutation common between the scientific literature and the clinical study appeared in the clinical data. The red line represents the average count of patients with a mutation for genotype D precore, which is 5.67 patients. The order of the mutations in the bar chart is based on the position of the nucleotides in the genes for genotype D according to the GenBank accession code AF121240.

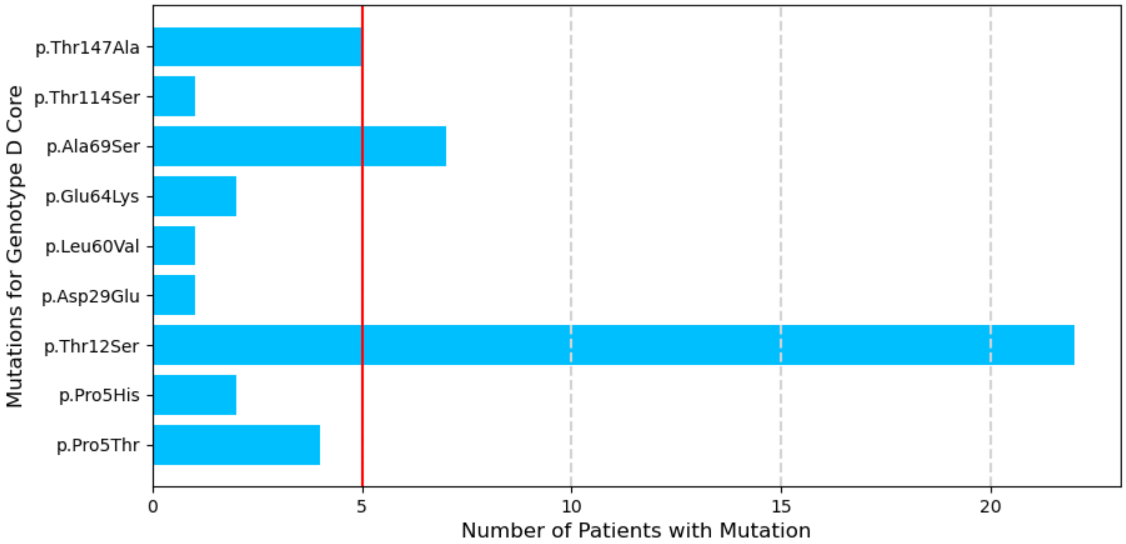

Supplementary Figure S7(h): Mutation hotspot map for HBV genotype D core (gene C) for amino acid variants. The counts for each mutation are based on the number of times a particular mutation common between the scientific literature and the clinical study appeared in the clinical data. The red line represents the average count of patients with a mutation for genotype D core, which is 5 patients. The order of the mutations in the bar chart is based on the position of the nucleotides in the genes for genotype D according to the GenBank accession code AF121240.

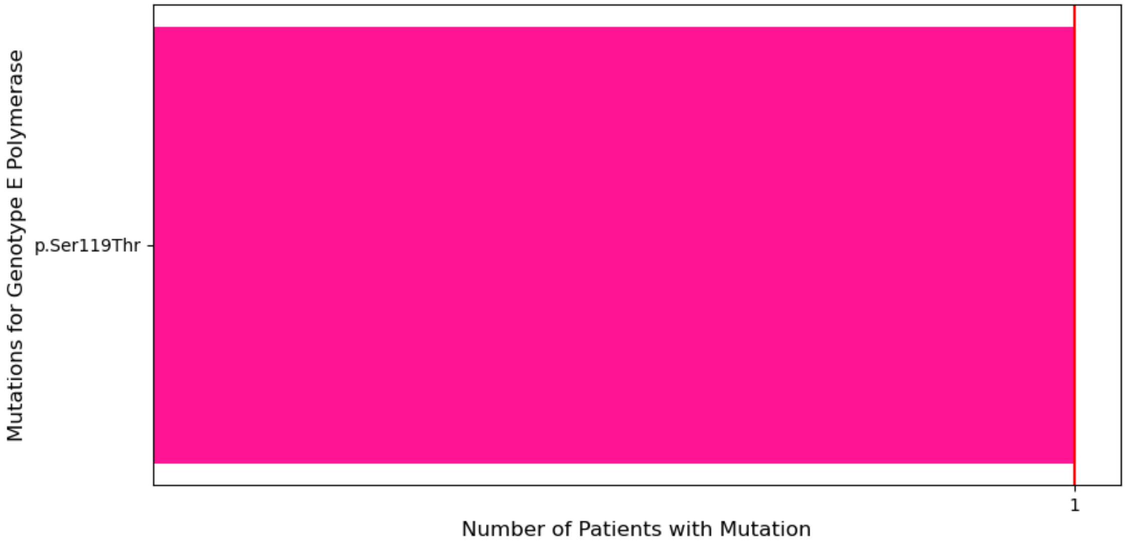

Supplementary Figure S8(a): Mutation hotspot map for HBV genotype E polymerase for amino acid variants. The counts for each mutation are based on the number of times a particular mutation common between the scientific literature and the clinical study appeared in the clinical data. The red line represents the average count of patients with a mutation for genotype E polymerase, which is 1 patient. The order of the mutations in the bar chart is based on the position of the nucleotides in the genes for genotype E according to the GenBank accession code AB032431.

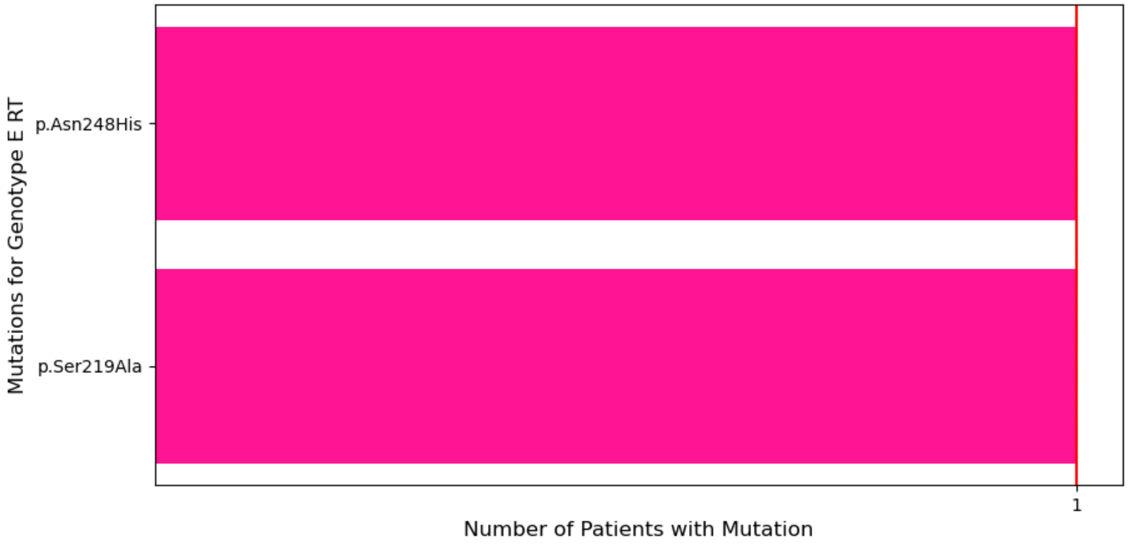

Supplementary Figure S8(b): Mutation hotspot map for HBV genotype E reverse transcriptase (RT) for amino acid variants. The counts for each mutation are based on the number of times a particular mutation common between the scientific literature and the clinical study appeared in the clinical data. The red line represents the average count of patients with a mutation for genotype E reverse transcriptase, which is 1 patient. The order of the mutations in the bar chart is based on the position of the nucleotides in the genes for genotype E according to the GenBank accession code AB032431.

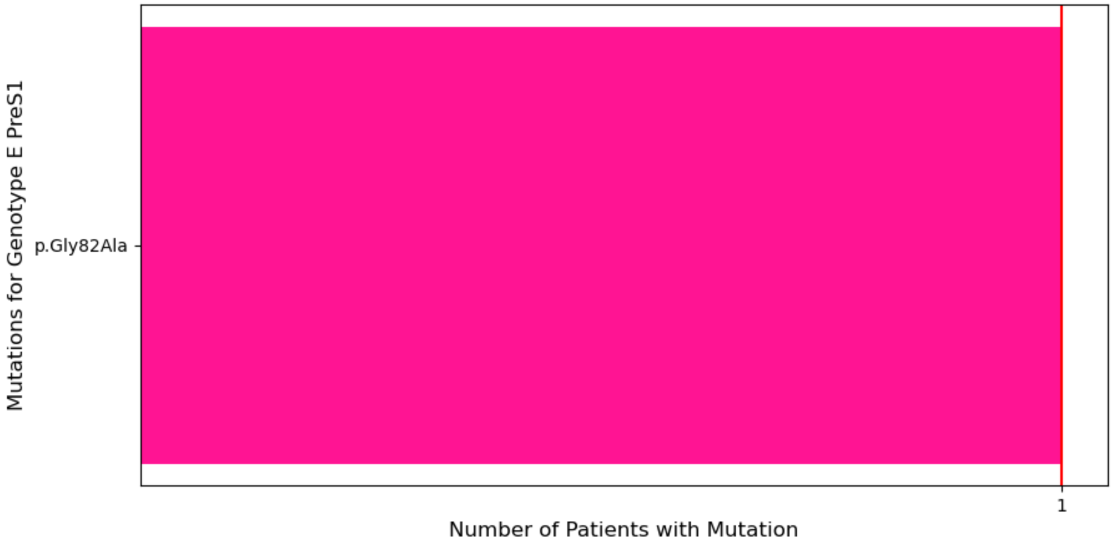

Supplementary Figure S8(c): Mutation hotspot map for HBV genotype E PreS1 (gene S) for amino acid variants. The counts for each mutation are based on the number of times a particular mutation common between the scientific literature and the clinical study appeared in the clinical data. The red line represents the average count of patients with a mutation for genotype E PreS1, which is 1 patient. The order of the mutations in the bar chart is based on the position of the nucleotides in the genes for genotype E according to the GenBank accession code AB032431.

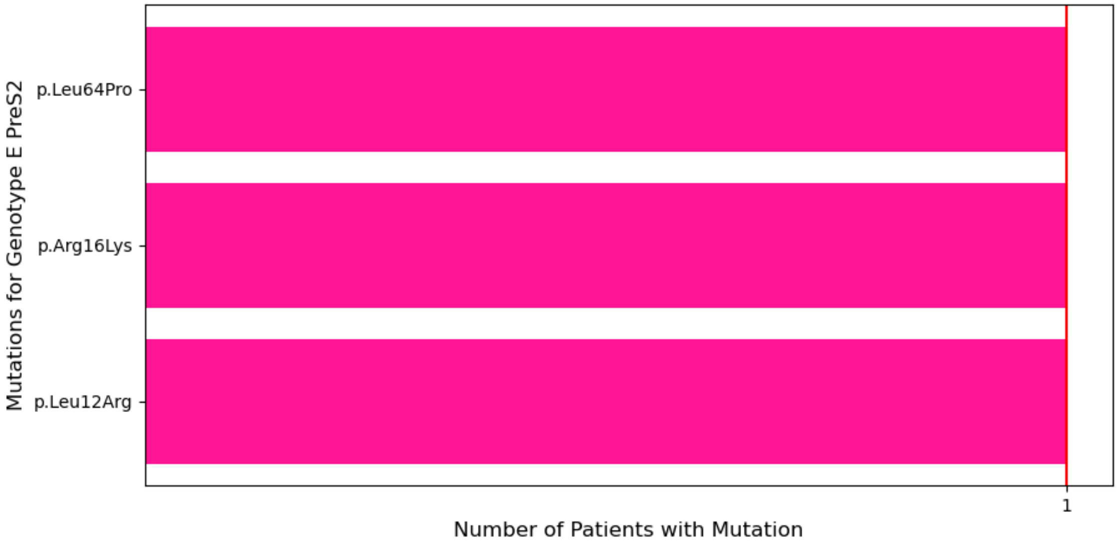

Supplementary Figure S8(d): Mutation hotspot map for HBV genotype E PreS2 (gene S) for amino acid variants. The counts for each mutation are based on the number of times a particular mutation common between the scientific literature and the clinical study appeared in the clinical data. The red line represents the average count of patients with a mutation for genotype E PreS2, which is 1 patient. The order of the mutations in the bar chart is based on the position of the nucleotides in the genes for genotype E according to the GenBank accession code AB032431.

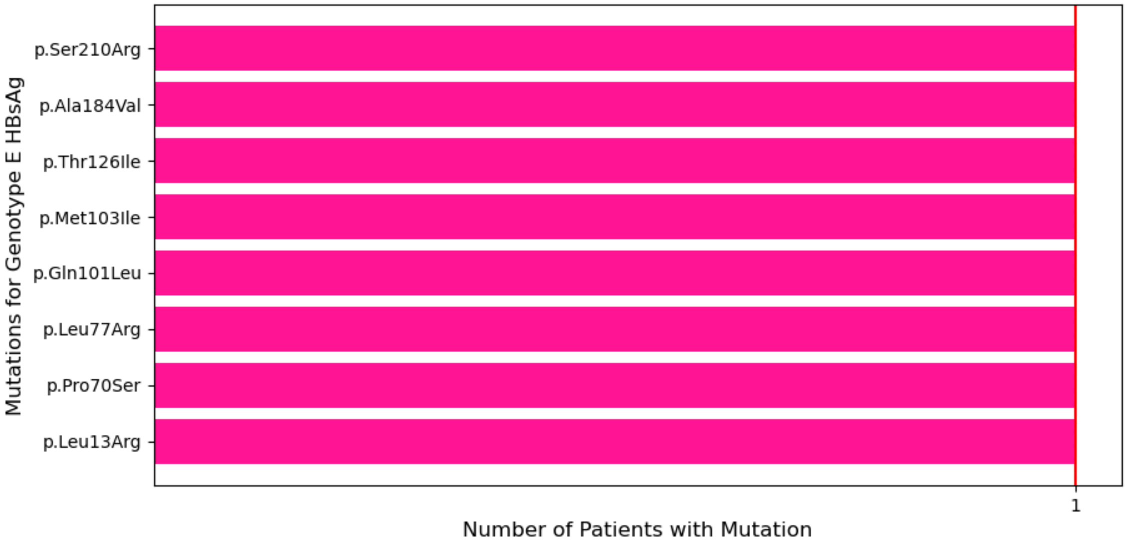

Supplementary Figure S8(e): Mutation hotspot map for HBV genotype E HBsAg (gene S) for amino acid variants. The counts for each mutation are based on the number of times a particular mutation common between the scientific literature and the clinical study appeared in the clinical data. The red line represents the average count of patients with a mutation for genotype E HBsAg, which is 1 patient. The order of the mutations in the bar chart is based on the position of the nucleotides in the genes for genotype E according to the GenBank accession code AB032431.

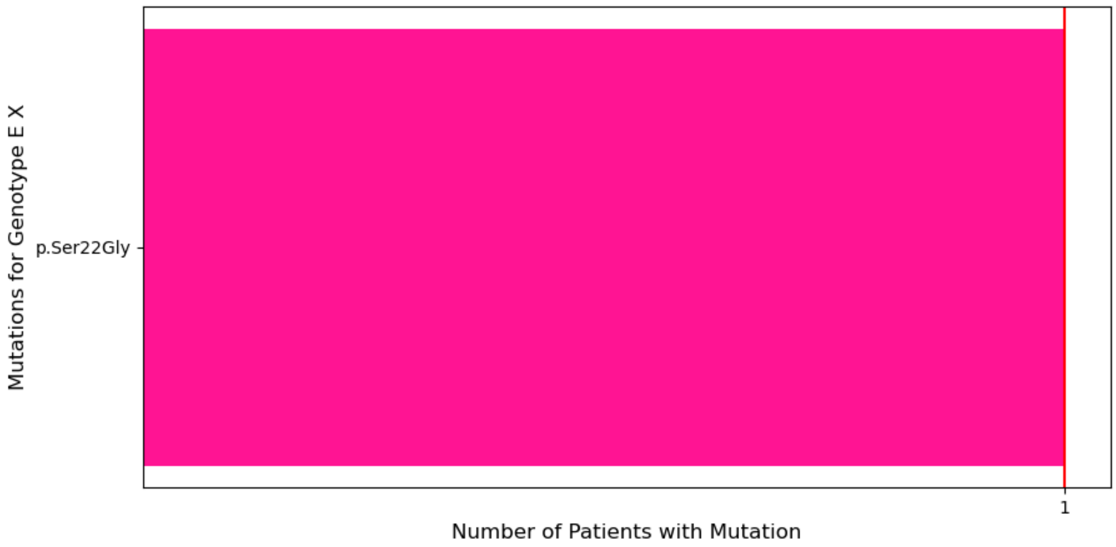

Supplementary Figure S8(f): Mutation hotspot map for HBV genotype E gene X for amino acid variants. The counts for each mutation are based on the number of times a particular mutation common between the scientific literature and the clinical study appeared in the clinical data. The red line represents the average count of patients with a mutation for genotype E gene X, which is 1 patient. The order of the mutations in the bar chart is based on the position of the nucleotides in the genes for genotype E according to the GenBank accession code AB032431.

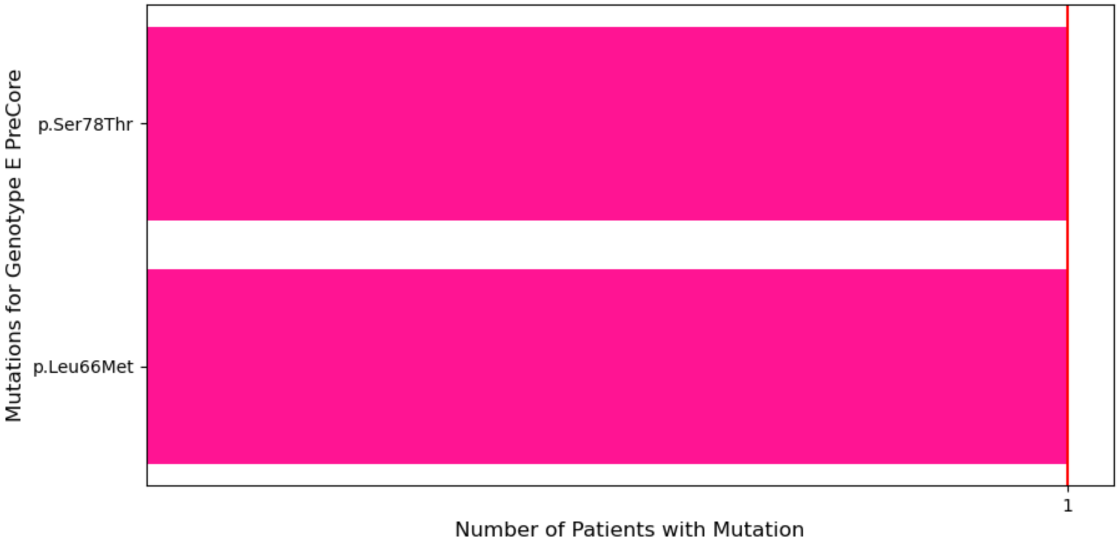

Supplementary Figure S8(g): Mutation hotspot map for HBV genotype E precore (gene C) for amino acid variants. The counts for each mutation are based on the number of times a particular mutation common between the scientific literature and the clinical study appeared in the clinical data. The red line represents the average count of patients with a mutation for genotype E precore, which is 1 patient. The order of the mutations in the bar chart is based on the position of the nucleotides in the genes for genotype E according to the GenBank accession code AB032431.

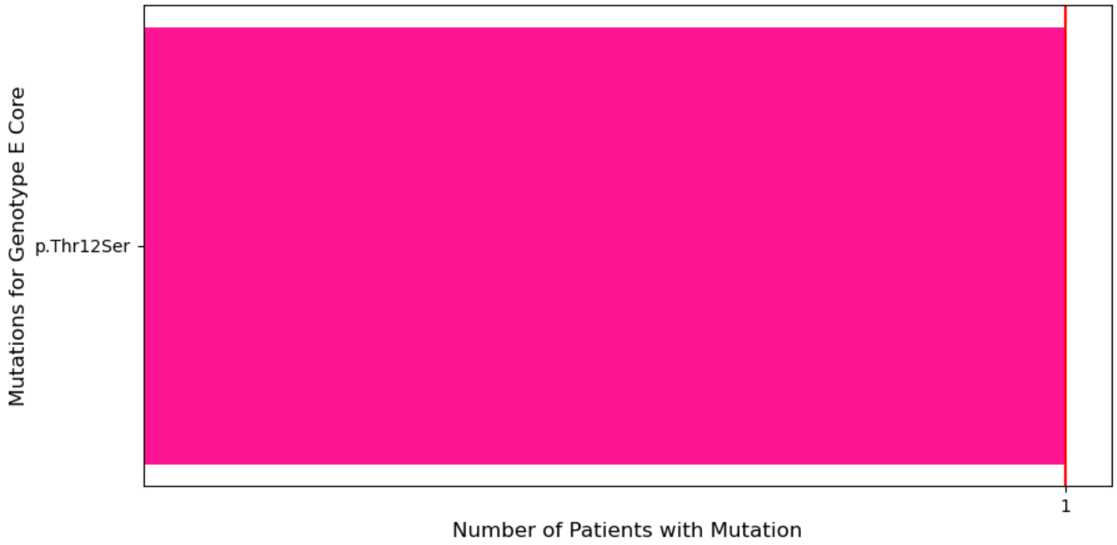

Supplementary Figure S8(h): Mutation hotspot map for HBV genotype E core (gene C) for amino acid variants. The counts for each mutation are based on the number of times a particular mutation common between the scientific literature and the clinical study appeared in the clinical data. The red line represents the average count of patients with a mutation for genotype E core, which is 1 patient. The order of the mutations in the bar chart is based on the position of the nucleotides in the genes for genotype E according to the GenBank accession code AB032431.

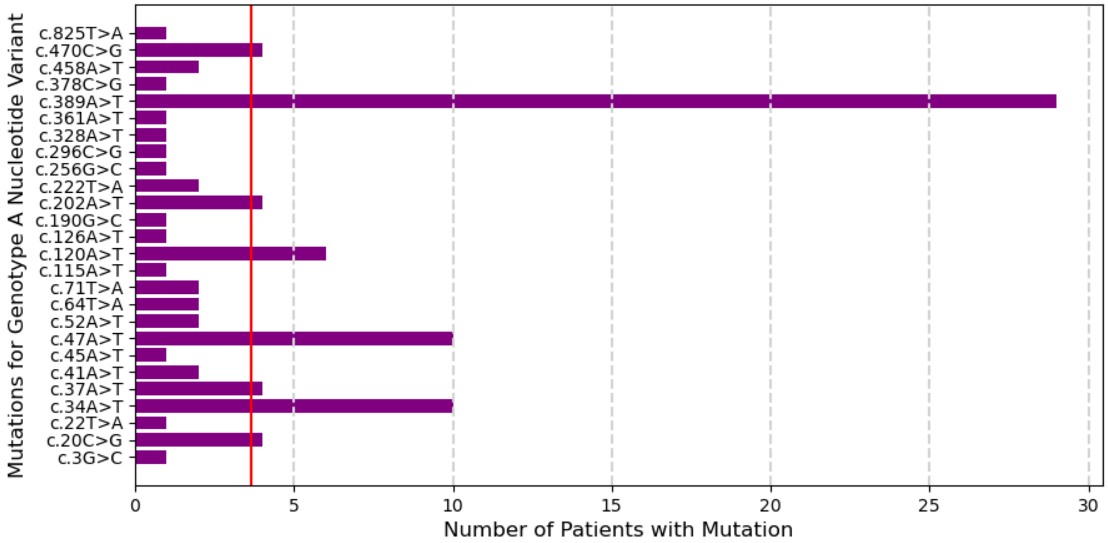

Supplementary Figure S9: Mutation hotspot map for HBV genotype A for nucleotide variants. The counts for each mutation are based on the number of times a particular mutation common between the scientific literature and the clinical study appeared in the clinical data. The red line represents the average count of patients with a mutation for genotype A nucleotide variants, which is 3.65 patients. The order of the mutations in the bar chart is based on the position of the nucleotides in the genes for genotype A according to the GenBank accession code AF090842.

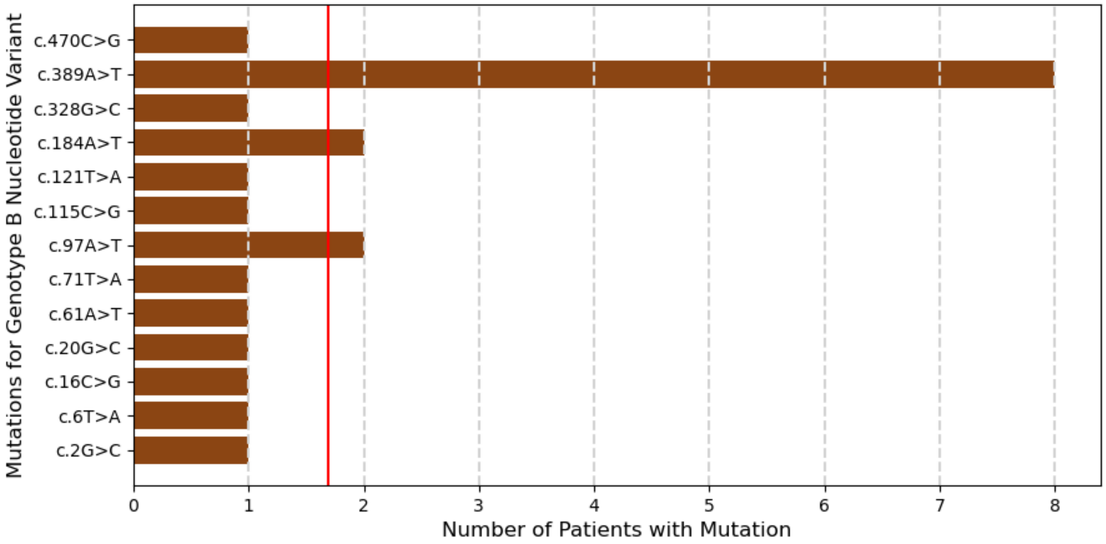

Supplementary Figure S10: Mutation hotspot map for HBV genotype B for nucleotide variants. The counts for each mutation are based on the number of times a particular mutation common between the scientific literature and the clinical study appeared in the clinical data. The red line represents the average count of patients with a mutation for genotype B nucleotide variants, which is 1.69 patients. The order of the mutations in the bar chart is based on the position of the nucleotides in the genes for genotype B according to the GenBank accession code AB033554.

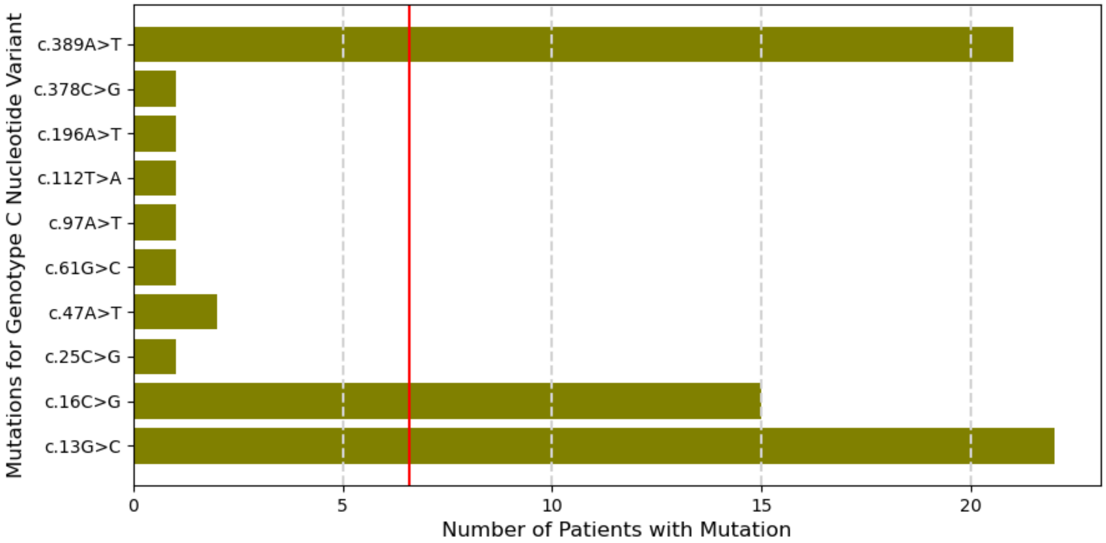

Supplementary Figure S11: Mutation hotspot map for HBV genotype C for nucleotide variants. The counts for each mutation are based on the number of times a particular mutation common between the scientific literature and the clinical study appeared in the clinical data. The red line represents the average count of patients with a mutation for genotype C nucleotide variants, which is 6.6 patients. The order of the mutations in the bar chart is based on the position of the nucleotides in the genes for genotype C according to the GenBank accession code AB033556.

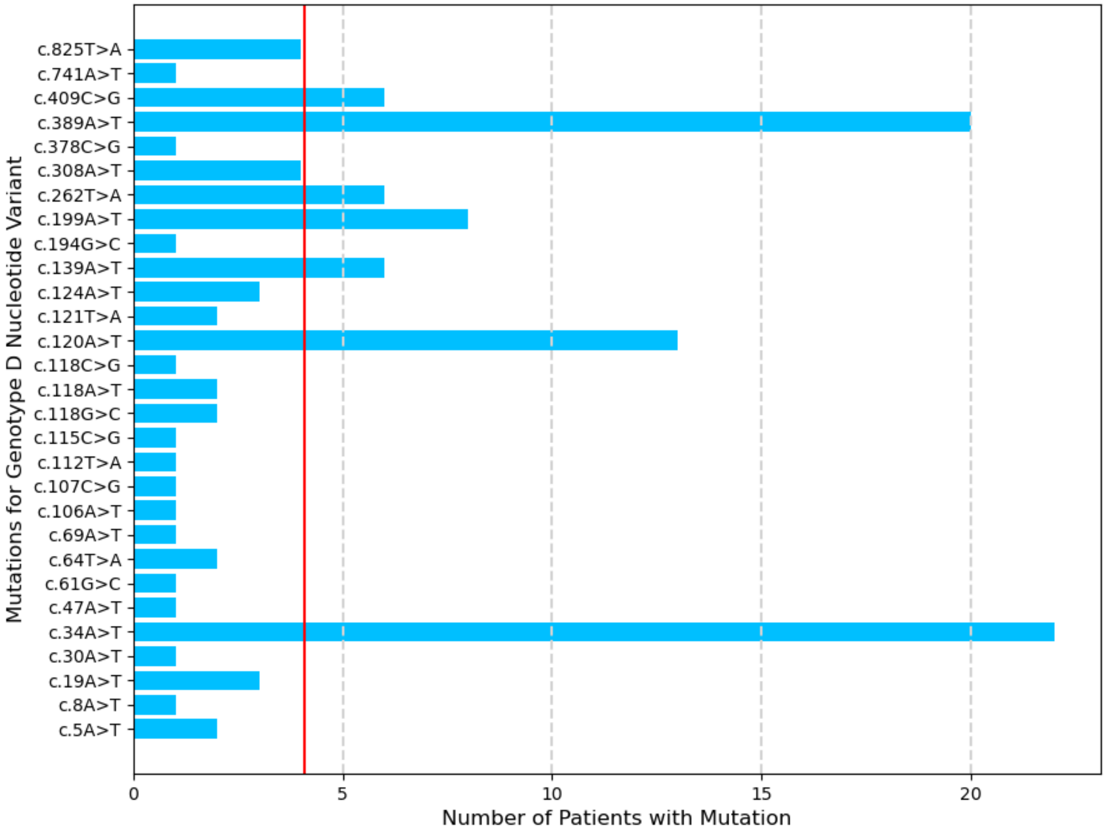

Supplementary Figure S12: Mutation hotspot map for HBV genotype D for nucleotide variants. The counts for each mutation are based on the number of times a particular mutation common between the scientific literature and the clinical study appeared in the clinical data. The red line represents the average count of patients with a mutation for genotype D nucleotide variants, which is 4.07 patients. The order of the mutations in the bar chart is based on the position of the nucleotides in the genes for genotype D according to the GenBank accession code AF121240.

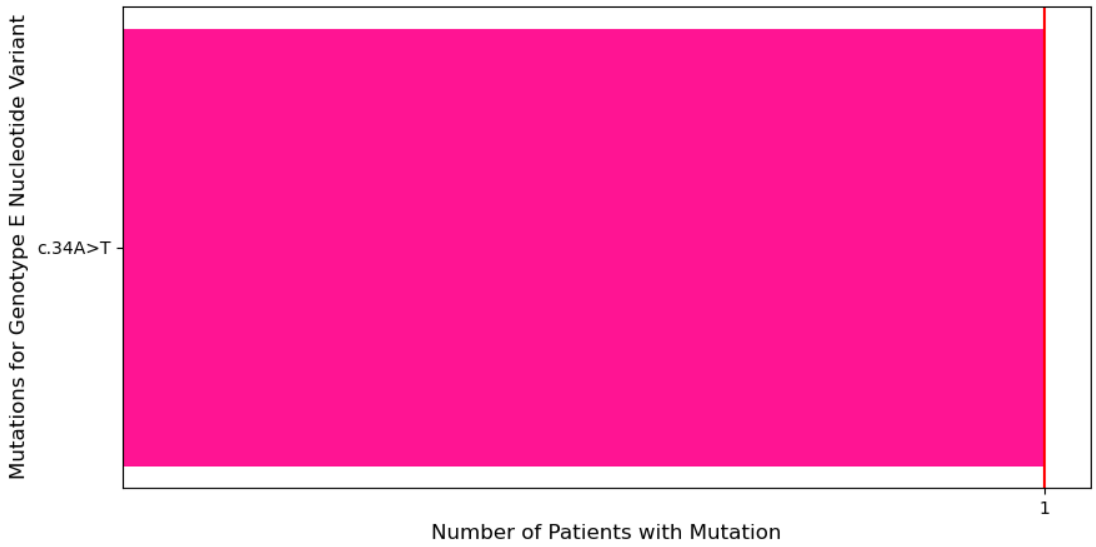

Supplementary Figure S13: Mutation hotspot map for HBV genotype E for nucleotide variants. The counts for each mutation are based on the number of times a particular mutation common between the scientific literature and the clinical study appeared in the clinical data. The red line represents the average count of patients with a mutation for genotype E nucleotide variants, which is 1 patient. The order of the mutations in the bar chart is based on the position of the nucleotides in the genes for genotype E according to the GenBank accession code AB032431.

Supplementary Table S1. Summary of hotspots for HBV genotype A for amino acid and nucleotide variants
(Note: * represents different mutations that were located at the same position twice).

Gene	Mean Number of Mutations	Position Number of Mutation Hotspots
Polymerase	6.50	F220, W501
Reverse Transcriptase	5.18	L129, I163, S213, L217, S219, D263, Q267
PreS1	4.00	S5, F141, N214, S219
PreS2	5.00	R16, S100*
HBsAg	3.31	E2, W36, K122, P127, Q129, M133, Y161, S204*, L209, S210
X	3.20	Y6
Precore	3.67	G29, S78
Core	3.60	P5*, T12, L60, T147
Nucleotide Variant	3.65	20C, 34A, 37A, 47A, 120A, 202A, 389A, 470C

Supplementary Table S2. Summary of hotspots for HBV genotype B for amino acid and nucleotide variants
(Note: * represents different mutations that were located at the same position twice).

Gene	Mean Number of Mutations	Position Number of Mutation Hotspots
Polymerase	1.33	K293
Reverse Transcriptase	3.18	D1, D134, S256
PreS1	2.33	F141, N214
PreS2	2.22	R122
HBsAg	1.72	E2, A5, K122, T126, M133*, A159, Y161, W182, S204, F220
X	1.00	P33, M103, T118
Precore	3.00	G29, L66
Core	1.50	I97, T147
Nucleotide Variant	1.69	97A, 184A, 389A

Supplementary Table S3. Summary of hotspots for HBV genotype C for amino acid and nucleotide variants
(Note: * represents different mutations that were located at the same position twice).

Gene	Mean Number of Mutations	Position Number of Mutation Hotspots
Polymerase	2.00	K293
Reverse Transcriptase	4.03	I91, D134, S223, I224, L269
PreS1	2.56	W4, S5, A81, I84, S96, F141, N214
PreS2	2.20	C1, S2, F151
HBsAg	4.52	R24, L77, Y100, Q101, I126*, G145, A184, Y221
X	1.33	P33
Precore	1.50	G29, I56, L66
Core	3.83	P5, L60, T147
Nucleotide Variant	6.60	13G, 16C, 389A

Supplementary Table S4. Summary of hotspots for HBV genotype D for amino acid and nucleotide variants
(Note: * represents different mutations that were located at the same position twice).

Gene	Mean Number of Mutations	Position Number of Mutation Hotspots
Polymerase	3.75	D16, L214
Reverse Transcriptase	4.38	N53, S78, L91, T128*, M129, Q149, V191, S213, Q215, S219, D263, Q267
PreS1	3.25	H17, R24, L54
PreS2	2.50	T7, H9, R16, I42, L54
HBsAg	2.91	E2, L109, P120*, P127, Y134, G159, W182, W196, P203, S204*, S207, S210, L213, F220
X	9.00	P33, T36
Precore	5.67	G29
Core	5.00	T12, A69
Nucleotide Variant	4.07	34A, 120A, 139A, 199A, 262T, 389A, 409C

Supplementary Table S5. Summary of hotspots for HBV genotype E for amino acid and nucleotide variants
(Note: * represents different mutations that were located at the same position twice).

Gene	Mean Number of Mutations	Position Number of Mutation Hotspots
Polymerase	1.00	S119
Reverse Transcriptase	1.00	S219, N248
PreS1	1.00	G82
PreS2	1.00	L12, R16, L64
HBsAg	1.00	L13, P70, L77, Q101, M103, T126, A184, S210
X	1.00	S22
Precore	1.00	L66, S78
Core	1.00	T12
Nucleotide Variant	1.00	34A

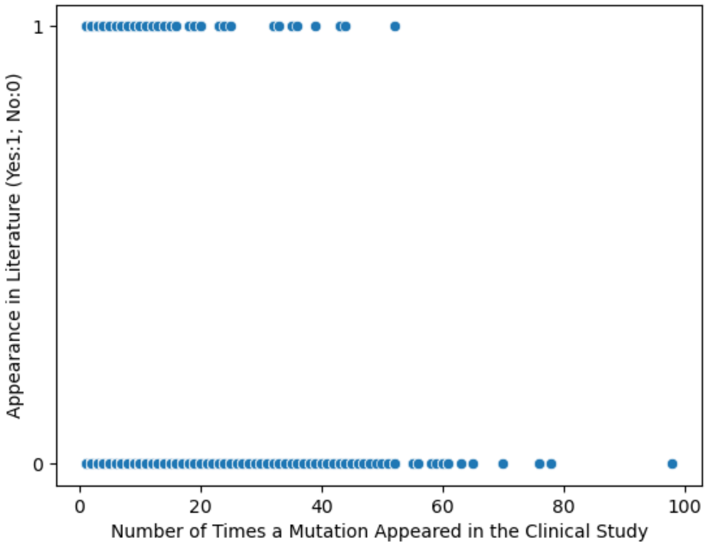

Supplementary Figure S14: Scatter plot to represent the count of a particular mutation found in the clinical study and whether any papers referred to that mutation (Yes: 1; No: 0) for amino acids.

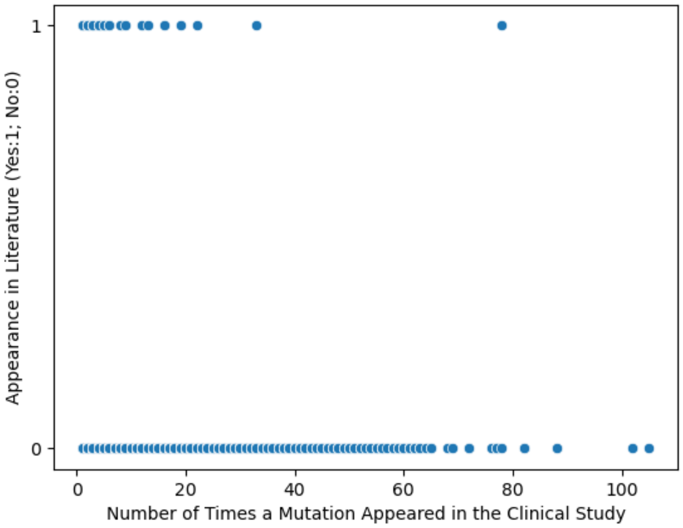

Supplementary Figure S15: Scatter plot to represent the count of a particular mutation found in the clinical study and whether any papers referred to that mutation (Yes: 1; No: 0) for nucleotides.

Appendix D: Probability of Drug-Resistance Mutations in the Literature and Clinical Study Counts

Supplementary Note S1: Linear model between the probability of appearing in the literature as drug-resistance-related and clinical study count.

We addressed the question of whether there is a correlation between how often drug-resistance mutations appear in the clinical study and in the literature. We created a simple linear model that assumes that the probability (p) of a clinical mutation to be mentioned as drug-resistance in the literature is dependent on the count of clinical samples available:

$$p = n * alpha$$

where:

- p is the probability of appearing in the literature as drug-resistance-related;
- n is the clinical study count; and
- alpha is a coefficient.

We ran a bootstrap simulation of 1000 iterations using log-likelihood maximization to compute alpha. For amino acids, the 95% confidence interval for alpha was:

$$[0.005906212760326079, 0.007312566823956289]$$

For nucleotides (NT), the 95% confidence interval for alpha was:

$$[0.0007410300736477067, 0.0012197424877538965]$$

Thus, the coefficient alpha is positive and greater than 0 but also quite small, particularly for NT. This linear correlation is something we would have expected but weaker than we would have expected.
